# Assessing concerns for the economic consequence of the COVID-19 response and mental health problems associated with economic vulnerability and negative economic shock in Italy, Spain, and the United Kingdom

**DOI:** 10.1371/journal.pone.0240876

**Published:** 2020-10-27

**Authors:** Cristiano Codagnone, Francesco Bogliacino, Camilo Gómez, Rafael Charris, Felipe Montealegre, Giovanni Liva, Francisco Lupiáñez-Villanueva, Frans Folkvord, Giuseppe A. Veltri

**Affiliations:** 1 Università degli studi di Milano, Milan, Italy; 2 Open Evidence Research, Barcelona, Spain; 3 Faculty of Information and Communication Science, Universitat Oberta de Catalunya, Barcelona, Spain; 4 Universidad Nacional de Colombia, Bogotá, Colombia; 5 Centro de Investigaciones para el Desarrollo, Bogotá, Colombia; 6 Tillburg School of Humanities and Digital Sciences, Tilburg University, Tillburg, The Netherlands; 7 Università degli Studi di Trento, Trento, Italy; University of North Texas Health Science Center, UNITED STATES

## Abstract

Many different countries have been under lockdown or extreme social distancing measures to control the spread of COVID-19. The potentially far-reaching side effects of these measures have not yet been fully understood. In this study we analyse the results of a multi-country survey conducted in Italy (N = 3,504), Spain (N = 3,524) and the United Kingdom (N = 3,523), with two separate analyses. In the first analysis, we examine the elicitation of citizens’ concerns over the downplaying of the economic consequences of the lockdown during the COVID-19 pandemic. We control for Social Desirability Bias through a list experiment included in the survey. In the second analysis, we examine the data from the same survey to predict the level of stress, anxiety and depression associated with being economically vulnerable and having been affected by a negative economic shock. To accomplish this, we have used a prediction algorithm based on machine learning techniques. To quantify the size of this affected population, we compare its magnitude with the number of people affected by COVID-19 using measures of susceptibility, vulnerability and behavioural change collected in the same questionnaire. We find that the concern for the economy and for “the way out” of the lockdown is diffuse and there is evidence of minor underreporting. Additionally, we estimate that around 42.8% of the populations in the three countries are at high risk of stress, anxiety, and depression, based on their level of economic vulnerability and their exposure to a negative economic shock.

## Introduction

In March 2020, COVID-19 was declared a pandemic by the World Health Organization [1] and has rapidly brought most countries into a strict enforcement of extreme social distancing. In particular, lockdown (that is, restrictions on movement, work and travel in order to prevent contagion by persons potentially exposed to the virus) was first introduced in China (23 January 2020) and later, in most of the world’s developed and developing countries. The lockdown has brought economies to a sudden and extreme halt: modern economies function on markets and markets require transactions and the mobility of factors. This mitigation strategy was justified by the evidence that social distancing works in flattening the curve of contagion and that it prevents health systems from being unable to cope [2–7]. A case in point is Italy, where in some regions (for example, Lombardy), the health system came close to collapsing after the country was reluctant to take measures to prevent a massive outbreak. The fear of having to deal with a similar situation contributed to convincing governments around the world that imposing such lockdown measures was necessary, given the notable reduction of the spread of the virus achieved in China [8–10].

Nevertheless, the Italian case presents several areas of doubt that are under investigation in order to determine whether or not they have had a causal impact: first, Italy has been reducing health expenditure significantly since the 2010 euro crisis [11]; second, it is one of the countries with the lowest intensive care units per 10,000 inhabitants [12]; third, it has one of the oldest populations in Europe [13]; and fourth, it is a society where the elderly have a high level of interaction with younger generations [14]. These have been reinforced by a delay in adjusting protocols within hospitals; for example, avoiding the admittance of patients exhibiting mild symptoms of COVID-19 [15], which has the potential to transform hospitals into clusters of contagion. This is reflected in the high mortality rate of medical personnel, in comparative terms (51 died before the end of March in Italy, [13]).

Designing a cost benefit effective mitigation strategy requires a consideration of the costs of measures that are introduced [16, 17]. In fact, there are strong arguments to suggest that the negative consequences of lockdown may have been underestimated and that they will have wide-reaching effects on various health dimensions, other than on the virus itself. First, it has been argued that the suicide rate, mental health, domestic violence, and the neglect of other health conditions (for example, cancers) may increase as a result of the lockdown and social distancing measures [18, 19], however, these are not considered in the daily communications on the number of deaths due to COVID-19. Second, there are concerns related to the negative consequences of income shocks; for instance, data from applications for unemployment insurance have increased exponentially in the US (reaching 33m according to the Department of Labor, and likely underestimated, [20]). We know from studies on scarcity that this may cause long-term problems in terms of cognitive resources and behavioural change [21], and may weaken social cohesion and increase the crime rate once society is out of lockdown and functioning normally again [22, 23]. Third, there is a fundamental argument that the current measures are driven by the precautionary principle and not by cost-benefit analysis. Although reasonable *a priori*, the precautionary principle is usually contested on two grounds: (a) if regulation is defended on the principle of the worst scenario, then a lack of regulation can be defended by the same argument when the consequences of strict regulations are potentially catastrophic; (b) the precautionary principle claims that fear should not be downplayed, even if the numbers do not agree, but this exposes the risk that *availability cascades* (the combination of availability heuristics and information cascade) dictate the agenda in public policy [24]. As an example, sub (a), one could argue against the lockdown, *ex ante*, because it could cause the worst economic crisis since the Great Depression, and sub (b) in favour of an indefinite extension of the lockdown, *ex post*, on the basis of the fear of a new outburst of COVID-19.

Current scientific research on COVID-19 is increasingly focusing on the medical, physical and social consequences of the crisis; showing that the virus is posing societal challenges across multiple dimensions. Within social sciences, the current research has focused on perception and attitudes [25], on the threat posed by COVID-19 related fake news [26, 27], and on designing nudges and other social and behavioural measures to support the COVID-19 pandemic response [28, 29], among the others. Unsurprisingly, evidence on the (mental health) side effects of the lockdown is largely missing, whereas in terms of contagion (reduction), data gathering has been wide-spread and systematic (almost) everywhere. Some evidence of the negative impact on mental health has been documented in China [30, 31], India [32], but mostly with convenience samples; yet, there is a consensus that mental health will be dramatically affected [33]. Given that the COVID-19 emergency has impacted developed countries before developing countries, and that the latter have stronger constraints in terms of savings, social policy and health infrastructure to face these problems, the assessment of the economic and health costs should be considered an immediate priority for public policy. There is also a hypothesis suggesting that the pandemic may last longer than 18 months and that periods of lockdown may need to be re-introduced (with second and potentially even further waves of the virus); this scenario also supports having enough evidence to make properly informed decisions [34, 35].

In this article, we intend to fill this void of evidence on the various side effects of the COVID-19 pandemic in the current literature by presenting the results of a multi-country (Italy, Spain and the United Kingdom [UK]) survey. This article is divided in two parts, based on separate analyses of the same data. In the first part, we elicit the level of concerns by citizens across three countries over the claim that the mitigation strategies used to contain COVID-19 have been neglecting or underestimating the magnitude of the economic consequences [36, 37]. In the second part, we attempt to map how these concerns for the economic consequences of the lockdown are related to mental health issues. In the multi-country survey, we assess the level of stress, anxiety and depression in the population as predicted by economic vulnerability (for example, low socio-economic background, low residential space, etc.), with the aim of identifying the potential size of mental health problems due to the side effects of the lockdown.

For the first part of this study, the outcome variables studied in this contribution are the level of support given to the following statements: “During the pandemic, the government should not only focus on preventing contagion but also on avoiding a major economic crisis”; and, “During the pandemic, the government should not only communicate to citizens what to do to adhere to the safety measures, but also clearly explain how it is planning the way out”. The first statement refers to the need to balance the losses in the domain of the pandemic and of the economy, as this need has been dismissed by part of the scientific community in favour of a unidimensional policy approach based on the precautionary principle [38]. The second statement refers to the need for transparent communication to allow households to plan their consumption, labour and investments during the year [18, 38].

If there is strong support for these two statements, we anticipate that such concerns will be reflected in various forms of stress associated with new sources of economic uncertainty and vulnerability, which we investigate in the second part of this study. Indeed, in the second part, the outcome variables are self-reported stress, anxiety, and depression.

Stress occurs when the level of stimuli exceeds a human body’s regulatory capacity. Anxiety is a reaction to stress, with feelings of worry, nervousness, or unease. Depression is a serious medical illness concerning feelings and emotions and is usually associated with sadness and loss of interest causing a lack of ability to function in individuals [39]. Stress, anxiety, and depression are used in this study to measure mental health [40].

In conducting these two analyses, we attempt to make three contributions in terms of estimating the side effects of the lockdown in response to COVID-19. First, we elicit support for the two statements related to the risk of neglecting the economic consequences. Since there may be social desirability bias (SDB) on this matter, we use a list experiment (included in the survey) to control for this. This is important because real support by citizens is fundamental to guarantee adherence to policy interventions, and because opposition to the current regulatory framework can be a predictor of the loss of social cohesion in the medium term.

Second, we estimate the extent to which the socio-economic background of a household can predict perceived stress, anxiety, and other mental health issues in relation to the current pandemic and its mitigation strategy. To estimate the dimension of mental health associated with the economic side effects of the lockdown, we use machine learning techniques (random forest) to predict the likelihood of being highly stressed, conditional on a number of critical factors; such as, organising home-schooling, having only a small living space, a low financial buffer stock, having suffered previous negative economic shocks, and so on. To provide some comparative quantification for this impact, we quantify through the same questionnaire the three components of exposure to COVID-19; namely susceptibility (the risk associated with the illness once contracted), vulnerability (the risk of exposure), and the behavioural response (people asking to be tested or contacting a doctor or health authorities due to COVID-19).

An additional contribution of this work is that it is a multi-country study of Italy, Spain, and the UK. These countries have a high number of deaths in common and are currently among the harshest hit in Europe. They have also adopted quarantine and lockdown measures, but with some difference in degree (for example, the UK first announced an alternative strategy based on herd immunity before later reverting to the lockdown strategy). Finally, the timing for the curve of contagion in the three countries has differed (the sequence being first Italy, second Spain and last the UK), and this gives us a variation in terms of length of exposure to lockdown. These features increase the external validity of the study.

Our first theoretical hypothesis is that experiencing lockdown and a lack of transparent communication on how to move in an orderly fashion towards a post-lockdown scenario increases the focus on the economic situation by citizens. This is consistent with the hypothesis that frustration and demoralization are exacerbated by a lack of commitment to keep the lockdown to as short a duration as possible, and that extensions may backfire [18]. Another potential channel is the scarcity mind-set: having to face negative economic shocks may increase the tendency of citizens to think in terms of opportunity cost and trade-off, becoming more focused on economic consequences [41]. A potential critique to the quest for transparency is that in presence of high polarization, opposition against the government will criticize the decisions regardless, and that citizens have different capabilities and resources to follow the scientific debate. However, we claim that transparency and accountability may play a role in reducing polarization.

Our second hypothesis is that adverse economic conditions predict worsened psychological wellbeing. Studies on the relationship between economic contraction and mental health have shown that the relationship between adverse economic experiences and depression is one of the most consistent findings [42, 43]. Participants in these studies who lost their jobs for reasons unrelated to health had a greater chance of experiencing depressive symptoms, even after controlling for workforce experience, mental ability, and socioeconomic variables. Indeed, not only job loss but also the type of work contract is associated with mental health issues. People who move from stable to inadequate employment demonstrate an increased risk of depression. In their review, [44] gathered evidence showing that people who experienced unemployment or impoverishment are at a greater risk of suffering from depression, alcohol abuse and suicide than people who did not. This problem is clearly aggravated for a household in a vulnerable situation since a low economic background offers less monetary and cultural resources to cope with adversity.

## Materials and methods

We submit a link to a random sample from a representative online panel (BdI Research’s Panel) in Italy, Spain, and the United Kingdom. We restrict participants to 18–75 years old. Although this is underestimating the impact on a subset of the population which is highly vulnerable to COVID-19, access to them through the online channel and the reliability of responses may be highly questionable. Data collection occurred between April 24—May 1, 2020. The study is preregistered on OSF (DOI:10.17605/OSF.IO/6XWE8). The English, Spanish and Italian versions of the questionnaire can be found in S1 File, Sections 1.1–1.3. In SOM, Section 1.4, we report population and sample proportions according to gender, age, and geographical residence; differences are minor. Given random sampling and the features of the original panel, our sample is representative of the 18–75 population with access to internet.

Ethics approval was obtained from the Institutional Review Board of the Universitat Oberta de Catalunya, the relevant documents of the IRB approval are available at the OSF page of the study. Approval includes the collection of data in the three countries, since they were recruited from the same online panel. All respondents provided informed consent.

In the first part of the questionnaire, we perform a list experiment, in the second part, participants answer a questionnaire.

A list experiment is a questionnaire design technique used to mitigate the respondent’s social desirability bias (SDB) when eliciting information about sensitive topics. With a large enough sample size, list experiments can be used to estimate the proportion of people for whom a sensitive statement is true. We measured the level of agreement with the following two statements:

- *During the pandemic*, *the government should not only focus on preventing contagion but also on avoiding a major economic crisis;*
- *During the pandemic*, *the government should not only communicate to citizens what to do to adhere to the safety measures*, *but also clearly explain how it is planning the way out*.

To prevent SDB from biasing the results, we considered four additional and unrelated statements, which are not the object of analysis, and we asked participants to state *how many* (rather than *which*) statements they agreed with.

We proceeded in the following way. We selected at random four subsamples of participants and asked them to perform the following tasks: the control group is presented with four statements, without using the controversial ones above, and is asked how many they agree with; treatment group one is presented with a set of five statements (four of the control and the first statement object of analysis) and is asked how many statements they agree with; treatment group two is presented with a set of five statements (four of the control and the second statement object of analysis) and is asked how many statements they agree with; last, treatment group three is asked direct questions; that is, after being presented with the full set of six statements, it is asked which statements they agree with.

As a result, comparing the average number of items selected in the control with the average number of items selected in treatment one (two) group, we detect the share of people who agree with the first (second) controversial statement. By comparing the share of people who agree with a controversial statement, as estimated through the list treatment, with the share of people who declared that they agreed with it (from the direct questions in treatment group three), we can estimate the SDB.

Assignment to treatments is as follows: 30% of the sample is randomly assigned to the control group, 30% to treatment group one, 30% to treatment group two, and 10% to treatment group three. The group with a direct question is smaller because the standard errors in direct response are much reduced than in the list experiment.

Uncontroversial items are: “Globalization has benefitted most of the population in the world”, “Immigration is a threat for our lifestyle”, “The health professionals are facing the largest risk in this pandemic”, “On important policy issues, the government should always follow the opinion of the experts”. Notice that we choose the first two and the fourth statements because people tend to have polarized opinions on them, and this guarantees that we are not facing ceiling effects. We add the third statement to ensure that we include a statement which is health-related, and that we do not prime participants too strongly towards reflecting on economic costs.

To analyse the list experiment, we followed [45], and used linear regressions, since we are not primarily interested in the identification of the impact of covariates. We used the following control variables, which are not causally affected by the treatment: a dummy for female, age, a dummy for marital status, a dummy for unemployed, household size, the number of children of school age, country dummies, income level, educational level, a dummy for homeownership, and the size of the house.

The post experimental questionnaire is standardized and includes characterization of socio-economic strata and health status. All the questions have been taken from Standardized Questionnaire from National Statistical Offices. Additionally, we measured stress, anxiety, and depression, and COVID19 exposure (susceptibility, vulnerability, and behavioral response).

We measured stress, anxiety and depression through an adapted version of the DASS-21 (Depression, Anxiety and Stress Scale– 21 Items; [46]) and of SASRQ (Stanford Acute Stress Reaction Questionnaire; [47]). The last questionnaire was created to measure acute reactions to stress, which means that the instrument is used to measure stress due to a specific stressor, not a general measure of stress [47]. Our questionnaire is close to the one used by the multi-country study of [25]. This is an eight-items scale, with answers from one to four. To estimate the relationship between the factors of economic vulnerability and exposure to negative economic shocks and stress, anxiety, and depression we used a random forest model, with bootstrapping, using 550 iterations. We used the following predictors in the model: household income, a dummy for unemployed, a dummy for homeownership, living space, household size, number of children of school age, financial buffer stock, negative events the occurred in the previous week, and change in income or earnings (SOM, Questionnaire, Q5, Q7, Q9, Q10, Q11, Q13, Q17a/b/c/d/e/f/h/i, Q27).

To project the results to the overall population, we post-stratified the results using gender, age (18–35; 36–55; 56–75) and residence (North; Centre; South; Isles for Italy, Madrid and Centre; Barcelona and West; North; Centre-East; South for Spain; East and Midlands, London, South; North; Scotland, Wales and Northern Ireland for the UK). Data for the post-stratification are taken from Eurostat.

Our measures for COVID19 exposure are the following ones. Susceptibility refers to how strong the impact is when an individual is affected by the virus. We already know from the literature that age, specific health problems and comorbidity (in particular Diabetes, Hypertension, Asthma, Cardiovascular Disease, Cancer according to [48–50]), and poor health conditions are all key factors that augment the negative consequences of contracting the disease. We collect this information through the questionnaire (SOM, Questionnaires Q18-21 and Q25). Vulnerability is the likelihood of getting the illness and is typically associated with communitarian exposure. We measure this component by eliciting the factors preventing full compliance with the quarantine (see SOM, Questionnaire, Q15). Finally, the behavioural response is elicited through questions on having contacted doctors or health authorities, or having sought to get tested (SOM, Questionnaires Q17g and Q24).

To summarize susceptibility, vulnerability and behavioural response, we performed principal component analysis. We assessed sampling adequacy using the Keyser-Meyer-Olkin criterion. In all cases, we retain the component based on the eigenvalues greater than one.

## Results

### List experiment

In total, 10,551 participants answered the questionnaire: 3,504 in Italy, 3,524 in Spain and 3,523 in the UK. We provide a set of statistical tests to assess the balancing of covariates (a dummy for female, age, a dummy for marital status, a dummy for unemployed, household size, the number of children of school age, country dummies, income level, educational level, a dummy for homeownership, and the size of the house) in S1 Table in S1 File. Covariates are not systematically different across the four groups, but we reject the null hypothesis for some of them. All of the covariates included in S1 Table in S1 File are used as controls in the regressions.

**Fig 1:**
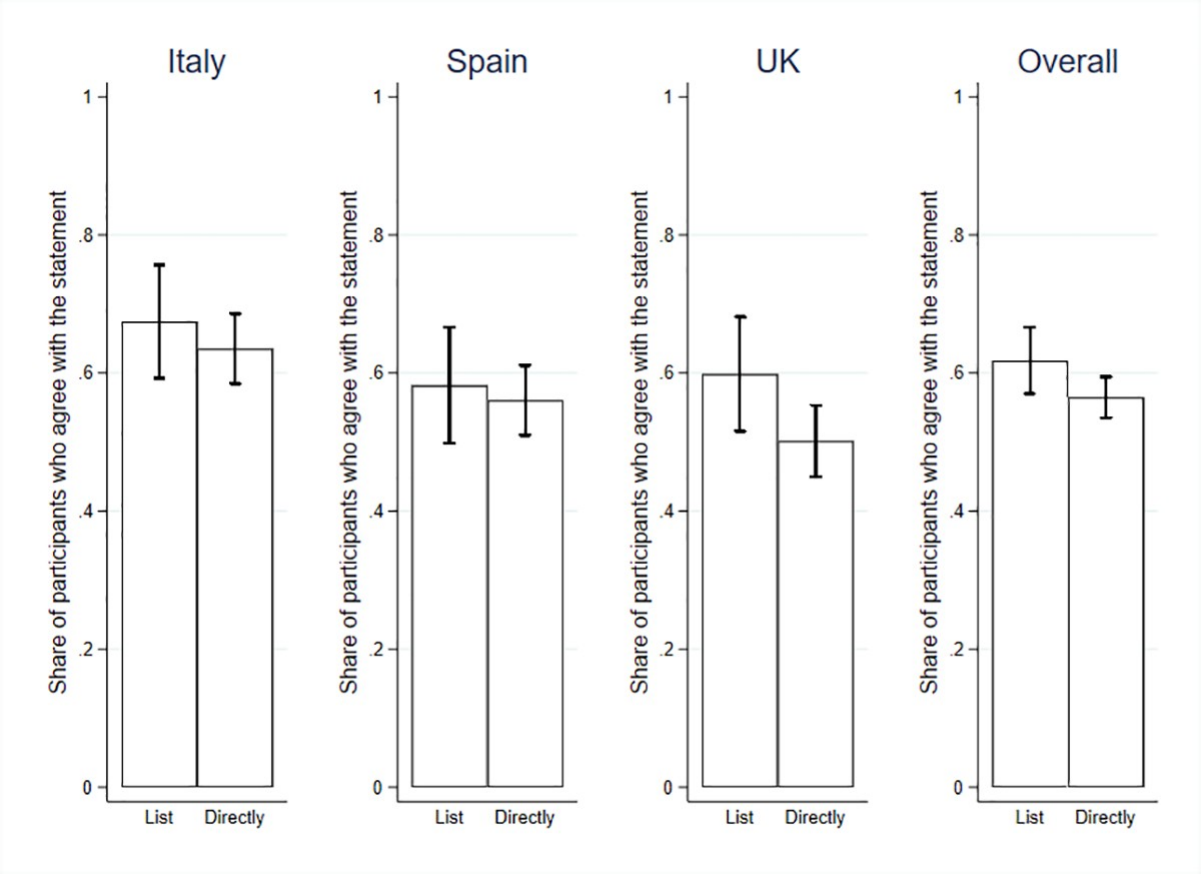
During the pandemic, the government should not focus only on preventing contagion but also on avoiding a major economic crisis.

**Fig 2:**
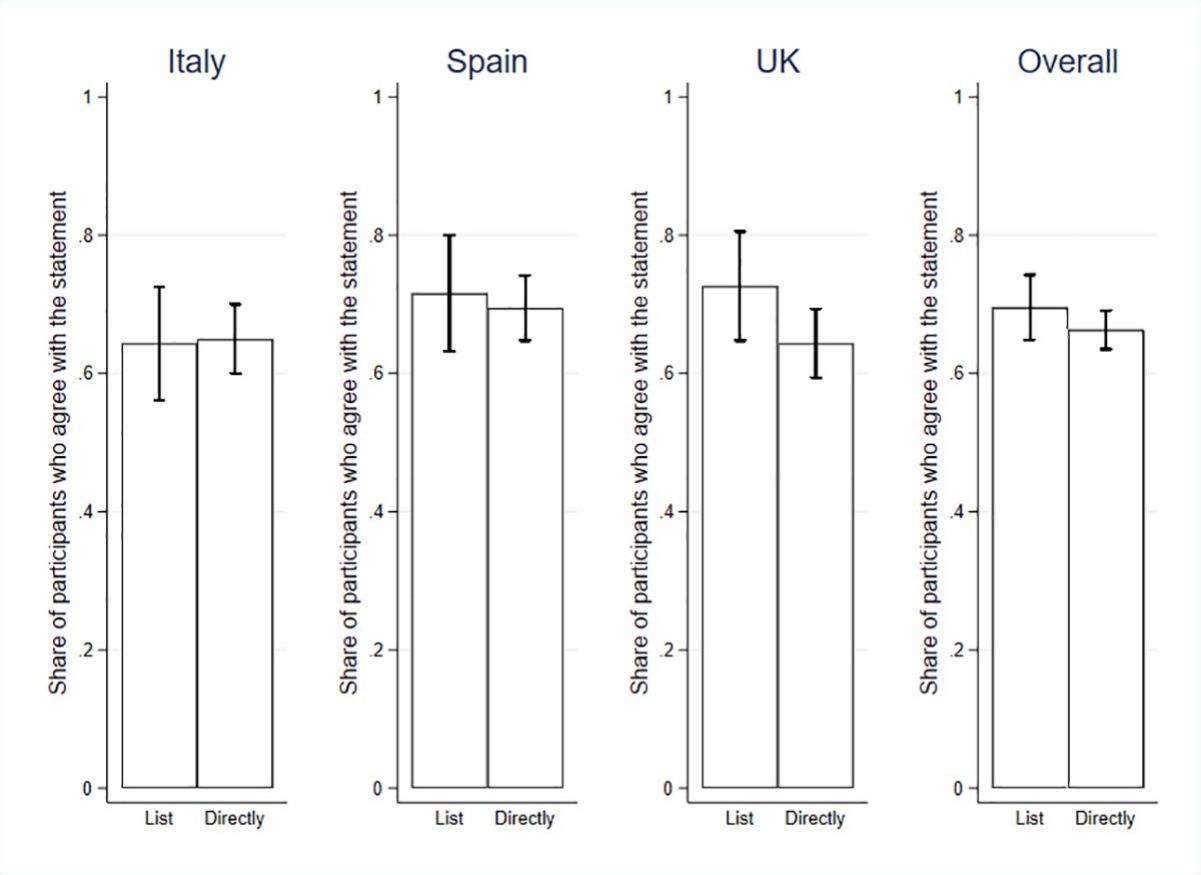
During the pandemic, the government should not only communicate to citizens what to do to adhere to the safety measures, but also clearly explain how it is planning the way out.

In Figs 1 and 2 below, we report the estimated support for the statements in the three countries and in the overall sample, and the extent of SDB, after controlling for a set of covariates. In the full sample, support for statement 1 is 62.09% estimated through the list experiment (t = 25.34, p < .000), and 56.48% by direct method (t = 244.62, p < .000). In the three countries, the results are as follows: in Italy, the average support revealed by the list experiment is 67.58% and 63.50% when asked directly; in Spain, the average support is 58.93% through the list method, and 56.03% through the direct method; and in the UK, it is 59.86% and 50.13%, respectively. Data support the presence of SDB (t = 480.10, p < .000). All of the supporting regressions are reported in the SOM, S2–S4 Tables in S1 File.

Similar results hold for statement 2, the support of which is 69.65% when estimated through the list experiment (t = 29.02, p < .000), and 66.29% by direct method (t = 294.78, p < .000). In the three countries, the results are as follows: in Italy, the average support revealed by the list experiment is 64.57% through the list method and 64.93% through the direct method; in Spain, the average support is 72.20% through the list method, and 69.43% through the direct method; and in the UK, it is 72.49% and 64.33%, respectively. The data fail to reject the absence of SDB (t = 387.10, p < .000). All of the supporting regressions are reported in the SOM, S2–S4 Tables in S1 File.

In S5 Table in S1 File, we report the frequencies for the number of items chosen, by treatment and country.

As argued by [45], the implicit assumptions in a list experiment are the absence of design effect and absence of liars. These assumptions state, respectively, that answers to uncontroversial statements do not change when controversial statements are added, and that participants do answer truthfully. The latter is untestable. The statistical procedure we use is one suggested by [45]. These tests are reported in the SOM, S6–S9 Tables in S1 File. We fail to reject the null hypotheses of the absence of design effect. These results suggest that our inferences from the list experiments have robust internal validity.

### Economic vulnerability and negative shocks predict mental health

Levels of stress, anxiety nd depression were measured through an adapted version of the DASS-21 (Depression, Anxiety and Stress Scale–21 Items; [46]) and of SASRQ (Stanford Acute Stress Reaction Questionnaire; [47]). Responses are elicited over a Likert scale from 1 to 4, where the items correspond to: rarely or none of the time (less than one day); some or a little of the time (1–2 days); occasionally or a moderate amount of time (3–4 days); most or all of the time (5–7 days).

In the Table 1 below, we report for each item the share of people who report having felt the symptoms at least one or two days in the previous week. The instrument is reliable (Cronbach’s alpha is 0.91).

**Table 1:** Stress, anxiety, and depression. Share of persons who felt the symptoms for at least one or two days of the previous week.

Item	ES	UK	IT	Total
Felt down, depressed or hopeless about the future	67.4%	56.9%	58.6%	60.9%
Felt little interest or pleasure in doing things	67.1%	59.8%	67.0%	64.6%
Felt nervous, anxious or on the edge	64.8%	59.9%	59.0%	61.3%
Had trouble falling or staying asleep, or sleeping too much	73.5%	64.4%	66.3%	68.1%
Felt bad about yourself—or that you are a failure or have let yourself or your family down	50.8%	48.1%	42.7%	47.2%
Had troubles concentrating on things	61.0%	57.6%	54.9%	57.8%
Had a physical reaction when thinking about the outbreak	40.0%	30.1%	32.5%	34.2%
Feeling tired or having little energy	66.9%	68.9%	70.3%	68.7%

The outcome variable is then normalized into an index on a 0.25 to 1 scale, averaging across the eight items. In Table 2 below, we report Spearman correlations with our socio-economic predictors (household income, a dummy for unemployed, a dummy for homeownership, living space, household size, number of children of school age, financial buffer stock, negative events that occurred in the previous week, and change in income or earnings). We use Spearman and not Pearson, in order to capture potential non-linearity and to minimize the effect of atypical values or response error. As suggested by our theoretical hypotheses, stress is highly correlated with economic vulnerability and exposure to a negative economic shock. According to Table 2, a higher income level is associated with lower stress (rho = -.04, p < .01), being unemployed is positively correlated with stress (rho = .09, p < .01), owning a house is negatively correlated (rho = -.08, p < .01), having a larger house space is negatively correlated (rho = -.10, p < .01), household size (rho = .10, p < .01) and especially having children of school age are positively correlated (rho = .12, p < .01), financial buffer stock (the time that bills can be covered after losing a job) is negatively correlated (rho = -.18, p < .01), having faced negative events is positively correlated (rho = .38, p < .01), as is having suffered job or income losses (rho = .19, p < .01). This is in line with our theoretical hypotheses.

**Table 2:** Relationship between economic vulnerability and stress, anxiety, and depression. Spearman correlation.

	Stress
Household income	-0.04***
Unemployed	0.09***
Home ownership	-0.08***
Living area	-0.10***
People in home	0.10***
Children in school	0.12***
Cover the bills	-0.18***
Stress events	0.38***
Income loss	0.19***

‘Household income’ is a categorical variable with the categories described in Q5 (SOM). ‘Unemployed’ is a dummy variable with a value of 1 if the respondent is in search of a job and 0 otherwise. ‘Homeownership’ is a dummy variable with the value of 1 if the respondent type of dwelling is owned and fully paid. ‘Living area’ is the respondent’s home useful living area in *m*^2^. ‘People in home’ is the household size. ‘Children in school’ reports how many children are of school age in the respondent’s household. ‘Cover the bills’ is a categorical variable with the categories described in Q16 (SOM). ‘Stress events’ is the sum of the response to the questions Q17-a), b), c), d), e), f), h) & i) (SOM). ‘Income loss’ is a dummy variable with the value of 1 if the respondent’s wage or earnings have been negatively affected after the COVID-19 outbreak and 0 otherwise.

*** p<0.01.

To quantify the extent of mental health issues associated with economic vulnerability and worsened economic conditions, we cannot rely on ex ante-ex post variation due to the lack of a pre-pandemic data point, and we do not have a source of exogenous variations of negative economic shocks and vulnerability. As a result, we compute the conditional probability of being under high stress, anxiety and depression given the independent variables in Table 1, being fully aware that we cannot claim causality. We estimate a standard random forest model between the outcome variable and the set of independent variables. We set the model using 550 iterations (trees) and regression as type of the decision tree. After computing the regression model, we estimate the share of those who are highly affected (outcome variable greater than or equal to .5) and project over the entire population, post-stratifying on age, gender and macro-region of residence. We compute that mental health problems predicted by the economic vulnerability and negative economic shock are 41.5% in Italy, 45.8% in Spain and 41.8% in the UK. Weighting for the population, this accounts for 42.8% in the three countries.

In the SOM (see S1 and S2 Figs and S10 Table in S1 File), we report the fit of the model (Spearman rank-order correlation coefficient = 0.9168, p = 0.0000), the out-of-bag error convergence, which stabilizes after 400 iterations at somewhere below 0.16, and the matrix of variable importance for each variable used when building the classifier. The results are highly correlated when we compare total sample and out of sample prediction with 15%, 25%, 33%, and 50% of the sample for learning (see SOM, Section 2, S11 and S12 Tables in S1 File). From the diagnostic tests, we conclude in favour of the robustness of the exercise.

Two clarifications are in order: during the pandemic, nurses and doctors, and their families, are facing the worst situation in terms of stress and anxiety. This is not duly reflected in our data since a targeted recruitment of medical personnel would be required and we did not want to disturb operations of hospitals during the crisis. Second, although a single data point cannot provide information on the counterfactual, it is still informative in terms of severity. In fact, clinical definition of depression and its psychopatological dimension in terms of intensity are based on the prevalence of a predefined number of symptoms over a time window [51]. This is equivalent to our approach.

One legitimate question is whether or not 42.8% represents many: the most obvious answer is to compare the exposure to mental health with the exposure to COVID-19, since the lockdown has been introduced to contain the risk of the pandemic.

There are three routes to estimate exposure to COVID-19: susceptibility, vulnerability, and behavioural response. We recall that we measure susceptibility through age, comorbidity (in particular Diabetes, Hypertension, Asthma, Cardiovascular Disease, Cancer), and self reported poor health conditions; vulnerability by eliciting the factors preventing full compliance with the quarantine. Finally, the behavioural response is elicited through questions on having contacted doctors or health authorities or having sought to get tested. We report descriptive statistics in the SOM, S13 to S15 Tables in S1 File.

Obviously, comparing each variable in each dimension with the predicted likelihood of being stressed conditional on vulnerability and shock is not highly informative, because we would get a difference response from different comparisons. To meaningfully summarize the information of the variables in each dimension of exposure to COVID-19, we perform a principal component analysis (that is, we represent each dimension -variable space- through a set of uncorrelated variables), which captures the largest part of its variability. In the three cases, sampling adequacy is acceptable (KMO sampling adequacy is 0.73 for susceptibility, 0.72 for vulnerability and 0.72 for behavioural response, see SOM, S16 Table in S1 File), and in all three cases the first component is the only one with an associated eigenvalue greater than one (2.5 for susceptibility, 50% of variance explained; 2.25 for vulnerability, 45% of variance explained; 2.22 for behavioural response, 55% of variance explained): thus, we can use the associated eigenvector (see SOM, S17 Table in S1 File) to build a score, increasing in the level of exposure.

**Fig 3:**
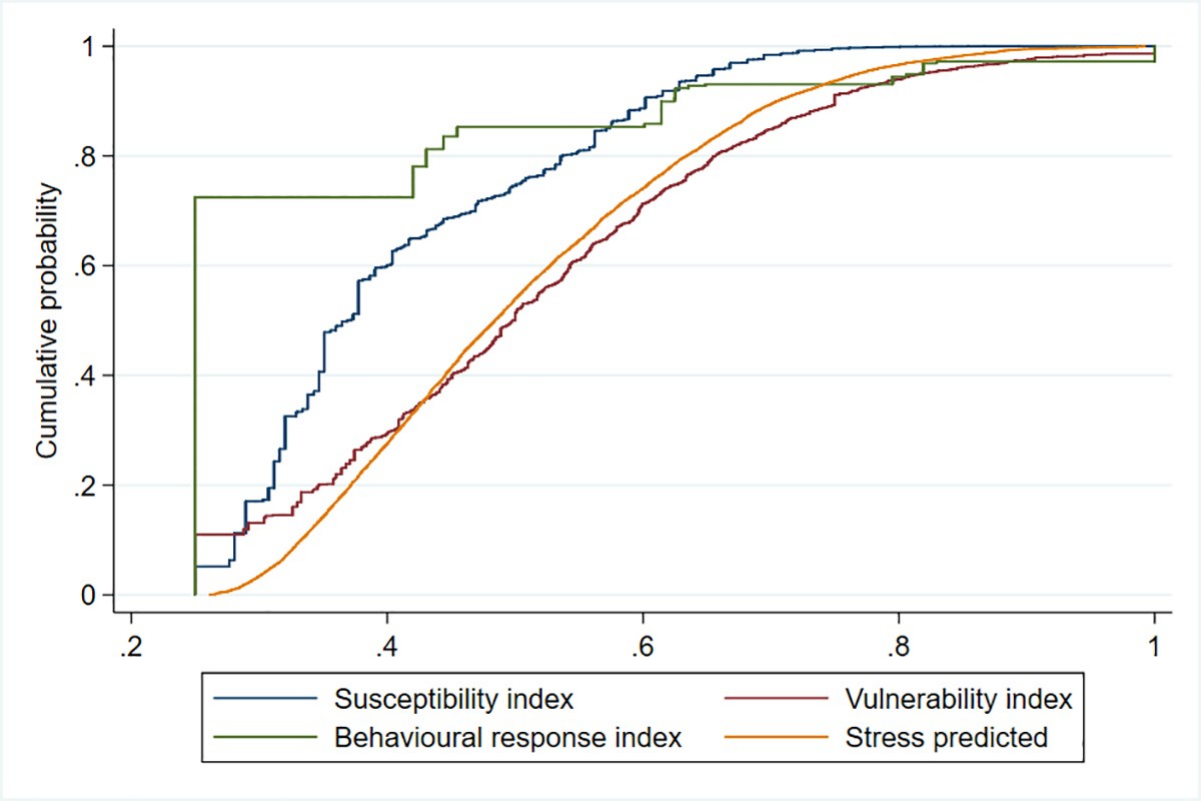
Comparing the cumulative distribution of exposure to susceptibility, vulnerability, behavioural response, and predicted stress conditional on economic vulnerability and negative economic shock.

As a result, we built four indexes, all scaled from 0.25 to one, three for exposure to COVID-19 and one for exposure to stress. Of course, defining a threshold in the first three scores (for example, .5 as in the stress case) would be completely arbitrary, since there is no clear cardinality in the scale of the score. To prevent arbitrary choices from biasing the comparison, we plot the cumulative density function; that is, for any value of the index *x*, the share of respondents who have *x* at most, P(x≤ *x*). Fig 3 plots the results and shows that stress stochastically dominates two out of the other three distributions at the first order. Formally, this can be written as P(stress≤ *x*) ≤ P(j≤ *x*) for all *x* between zero and one, and for j = susceptibility, vulnerability and behavioural response. Graphically, this can be seen from the fact that the distribution of predicted stress stands to the right of the susceptibility and behavioural change, but almost coincide with that of vulnerability (results are robust to the choice of the training sample, see SOM, S3 Fig in S1 File). In simple words, for any level of the score from 0.25 to 1, the likelihood of observing more people exposed above the selected level at least equal for stress than for any other measure of exposure to COVID-19.

## Discussion

In this study, we collected and analysed data from three European countries (Italy, Spain, and the United Kingdom), which have been under lockdown due to the recent outbreak of the COVID-19 pandemic that began in China in late 2019. The aim of the study is to measure the support for two statements related to the importance of the economic consequences of mitigation strategies and to measure the consequences of the lockdown in terms of mental health, as predicted by economic vulnerability and negative economic shocks. The hypotheses are that economic depression induces more worry for citizens regarding their economic situation, and that sharp negative economic shocks worsen psychological wellbeing.

This study shows a prevalence of concern for the unidimensional policy orientation during the pandemic on protecting the current healthcare systems, by putting aside other important elements of societies currently hit by the COVID-19 crises, which is in line with recent other studies [52, 53]. In particular, we found sizable support for a more traditional view of government as minimizing a loss function defined over different problems, health and the economic crisis, in this case. We also found general support for the idea that governments should communicate a coherent way out of the crisis: from an economic point of view, this involves asking the government to commit to certain long-term objectives to allow a household to reasonably protect itself from uncertainty and achieve a more reasonable solution in terms of consumption smoothing and investment (the same applies for businesses and companies). Most countries have stressed that it is imperative to delay and flatten the curve of the pandemic in order to ensure that national health services can cope with the resulting situation, and indeed, most governments have asserted that their responses to the COVID-19 pandemic are based on evidence and expert modelling. Nevertheless, different scientists have arrived at different conclusions based on the same evidence: small differences in assumptions can lead to large differences in modelling predictions, as can be seen in the different regulations implemented by EU Member States, and beyond [38].

Another important finding of this study is that economic vulnerability is associated with a strong risk of stress and worsening mental health. We estimated that around 42.8% of the population is at risk because of the negative shocks and the conditions of economic vulnerability in the developed countries in which we have conducted our study. This suggests that in developing countries, which must still face the worst part of the health emergency and the economic consequences of potential governmental regulations, the consequences may be even worse. The pattern we encountered is similar across the three countries we study (Italy, Spain and the UK), even though the timing of the quarantine and the initial strategy by the government in each country has differed, with the UK first announcing the intention to pursue herd immunity, but then also rapidly shifting towards standard isolation measures. Moreover, these results have strong external validity because of the representativeness of the sample in the three countries.

Our results have additional implications: while the epidemiological literature has expended a great deal of effort in profiling citizens according to their COVID-19 risk, our article suggests that a similar task can be successfully conducted in order to maintain societal resilience to mental health problems [54] and the resilience of the health system [55, 56].

One of the strengths of the current study is that we have assessed the differences between the three European countries studied, as they have all been significantly affected by COVID-19, while there are significant differences in each of their regulatory interventions. Second, considering the wide variety of studies published on this topic, we would argue that a deeper analysis on the effects of the regulatory interventions on mental health within society is missing but highly necessary in order to better understand the effects of these regulations. Third, we have collected data from a large group of representative citizens, thereby being able to generalize the outcomes.

Naturally, our study has limitations. First, although we have external validity within the three countries, we cannot claim generalizability to Europe or the world. This is somewhat unavoidable in the current situation in which there are environmental conditions that are important, as reflected in the heterogeneity in the death rate, but whose role is not fully understood.

Another limitation is due to the self-reported measurement levels of mental health. Although highly reliable, these questions do not represent medical data. However, autoptic recognition of mental health is a necessary step in providing a medical assessment, and as a result, it could be noisier, but it is unlikely to be biased in the opposite direction with respect to more clinical measurements. Additionally, we used a validated scale.

Third, the comparison between the exposure to stress and the exposure to COVID-19 is subject to critique. Although stochastic dominance is a conservative test the latter have been obtained through a filter (principal component analysis). This suggests that there may be some non-linear combination of the underlying variables (that is, some way in which to aggregate the information into a single dimension), for which the proposition no longer holds.

As has been argued, mental health could be the next threat to our societies, based on the conditions of the mitigation strategies and the hard-hitting economic depression that will likely follow. Mapping this evolution in terms of the population at risk, providing creative policy solutions that do not compromise the results achieved in terms of flattening the curve of contagion, and maintaining the resilience of our health system should be included among the future research priorities. Based on these results, it is also important for countries that are behind in controlling the spread of the virus, or vis-a-vis future outbreaks, to design ex ante more balanced mitigation strategies so as to prevent some of the side effects of lockdown and quarantine manifesting themselves.

## Supporting information

S1 File Supplementary online material. Includes Section S1.1 Questionnaire in English, Section S1.2 Questionnaire in Spanish, Section S1.3 Questionnaire in Italian, Section S1.4 Sample proportions. Section S2. Supplementary Statistical Analysis: S1–S17 Tables and S1–S3 Figs.
(DOCX)
