# Supplementary material for: Assessing concerns for the economic consequence of the COVID-19 response and mental health problems associated with economic vulnerability and negative economic shock in Italy, Spain, and the United Kingdom
Source: PLoS One. 2020 Oct 27;15(10):e0240876. doi: 10.1371/journal.pone.0240876 (PMC7591048; doi:10.1371/journal.pone.0240876)
Supplement: S1 File — Includes Section S1.1 Questionnaire in English, Section S1.2 Questionnaire in Spanish, Section S1.3 Questionnaire in Italian, Section S1.4 Sample proportions. Section S2. Supplementary Statistical Analysis: S1–S17 Tables and S1–S3 Figs. (DOCX) [file pone.0240876.s001.docx]

# Assessing concerns for the economic consequence of the COVID-19 response and mental health problems associated with economic vulnerability and negative economic shock in Italy, Spain, and the United Kingdom

# Supplementary Online Materials

*Cristiano Codagnone, Francesco Bogliacino, Camilo Gómez, Rafael Charris, Felipe Montealegre, Giovanni Liva, Francisco Lupiáñez Villanueva, Frans Folkvord, Giuseppe Veltri*

**Table of content**

[Assessing concerns for the economic consequence of the COVID-19 response and mental health problems associated with economic vulnerability and negative economic shock in Italy, Spain, and the United Kingdom 1](#_Toc48845756)

[Supplementary Online Materials 1](#_Toc48845757)

[**Section S1.1 Questionnaire in English** 3](#_Toc48845758)

[**Section S1.2 The Questionnaire in Spanish** 11](#_Toc48845759)

[**Section S1.3 The questionnaire in Italian** 20](#_Toc48845760)

[**Section S1.4 Sample proportions** 28](#_Toc48845761)

[**Section S2. Supplementary Statistical Analysis** 29](#_Toc48845762)

[**Table S 1** Balancing of the covariates 29](#_Toc48845763)

[**Table S 2** Statement “During the pandemic, the government should not focus only in preventing contagion but also in avoiding a major economic crisis” 30](#_Toc48845764)

[**Table S 3** Statement “During the pandemic, the government should not only communicate citizens what to do to adhere to the safety measures, but also explain clearly how it is planning the way out” 32](#_Toc48845765)

[**Table S 4** Direct response 34](#_Toc48845766)

[**Table S 5** Frequencies – list experiment 36](#_Toc48845767)

[**Table S 7** Test for design effects - treatment 1 38](#_Toc48845768)

[**Table S 8** Test ceiling and floor effects – treatment 2 39](#_Toc48845769)

[**Table S 9** Test for design effects - treatment 2 40](#_Toc48845770)

[**Fig S 1** Spearman rank-order correlation between stress and conditional prediction 41](#_Toc48845771)

[**Fig S 2** Out-of-bag error (OOB error) convergence 42](#_Toc48845772)

[**Fig S 3** Comparing the cumulative distribution of exposure to susceptibility, vulnerability, behavioural response, predicted stress conditional (50% of training sample) and predicted stress conditional on economic vulnerability and negative economic shock. 43](#_Toc48845773)

[**Table S 10** Variable importance – random forest algorithm 44](#_Toc48845774)

[**Table S13** Susceptibility to COVID-19 47](#_Toc48845775)

[**Table S 14** Vulnerability to COVID-19 48](#_Toc48845776)

[**Table S 15** Behavioural Response to COVID-19 49](#_Toc48845777)

[**Table S 16** Principal component analysis - Kaiser–Meyer–Olkin (KMO) 50](#_Toc48845778)

[**Table S 17** Eigenvectors principal component analysis 51](#_Toc48845779)

##

## **Section S1.1 Questionnaire in English**

**LIST EXPERIMENT**

Control

Below you will find a list of statements on which some people agree and others disagree. Please tell us how many of them you agree with. We do not need to know WHICH ones you agree with, just HOW MANY.

1. Globalization has benefitted most of the population in the world
2. Immigration is a threat for our lifestyle
3. The health professionals are facing the largest risk in this pandemic
4. On important policy issues, the government should always follow the opinion of the experts

Treatment one

Below you will find a list of statements on which some people agree and others disagree. Please tell us how many of them you agree with. We do not need to know WHICH ones you agree with, just HOW MANY.

1. Globalization has benefitted most of the population in the world
2. Immigration is a threat for our lifestyle
3. The health professionals are facing the largest risk in this pandemic
4. On important policy issues, the government should always follow the opinion of the experts
5. During the pandemic, the government should not only focus on preventing contagion but also on avoiding a major economic crisis.

Treatment two

Below you will find a list of statements on which some people agree and others disagree. Please tell us how many of them you agree with. We do not need to know WHICH ones you agree with, just HOW MANY.

1. Globalization has benefitted most of the population in the world
2. Immigration is a threat for our lifestyle
3. The health professionals are facing the largest risk in this pandemic
4. On important policy issues, the government should always follow the opinion of the experts
5. During the pandemic, the government should not only communicate to citizens what to do to adhere to the safety measures, but also clearly explain how it is planning the way out

Treatment three

Below you will find a list of statements on which some people agree and others disagree. Please tell us WHICH ones of them you agree with.

1. Globalization has benefitted most of the population in the world
2. Immigration is a threat for our lifestyle
3. The health professionals are facing the largest risk in this pandemic
4. On important policy issues, the government should always follow the opinion of the experts
5. During the pandemic, the government should not only focus on preventing contagion but also on avoiding a major economic crisis.
6. During the pandemic, the government should not only communicate to citizens what to do to adhere to the safety measures, but also explain clearly how it is planning the way out
7. How old are you?

__ years old

1. What is your sex?
   1. Female
   2. Male
   3. Other
2. What is the highest level of education you have completed?
   1. Primary school or less
   2. High school
   3. Some years of university (not completed)
   4. University degree completed
   5. Post-graduate (master, PhD, other)
3. What is your marital status?
   1. Single (never married)
   2. Married or in civil union
   3. Divorced or Widowed
4. What is your household (yearly) income?
   1. 9.999 Euro or below
   2. 10.000 Euro – 29.999 Euro
   3. 30.000 Euro – 49.999 Euro
   4. 50.000 Euro – 149.999 Euro
   5. 150.000 Euro or above
5. Which of the following best describe the area of your primary residency?
   1. Urban
   2. Suburban
   3. Rural
6. Which of the following situations best describes your current labor market status?
   1. Employed
   2. In search of job
   3. Student
   4. Retired
   5. Other (no work/no search/no study, housekeeper, disabled non-working person)
7. Which of the following occupations best describes your current prevalent activity?
   1. DIRECTORS: Directors, Executives, Directors of a company
   2. BUSINESSMEN, HOLDERS OF ACTIVITIES: Entrepreneurs, small companies or holders of activities
   3. INTELLECTUAL PROFESSIONALS, SCIENTISTS: Physicians, Chemists, Statisticians, Computer Scientists, Engineers, Architects, Biologists, Veterinarians, Pharmacists, Doctors, Dentists, Specialists in management, commercial and banking sciences, Lawyers, Solicitors, Notaries, Magistrates, University Professors (ordinary and associate), Specialists in economic, sociological, psychological, artistic, political, philosophical and literary sciences, Journalists
   4. TEACHERS: Upper and lower secondary school teachers
   5. TECHNICAL PROFESSIONALS: Physical and Chemical Technicians, Stock and Exchange Brokers, Commercial Agents, Representatives, Aircraft Pilots and Civil Aviation Technicians, Photographers, Nurses, Midwives, Dieticians, Hygienists, Paramedics, Insurance Agents, Experts, Designers, Computer Social Workers, Civil Construction Engineers, Web Operators, Programmers
   6. EMPLOYEES: Administrative employees, secretarial staff, accountants, employees in direct contact with the public, cashiers, counter employees
   7. TRADERS OR SERVICES: Wholesalers and managers of wholesale and retail sales, Office workers and similar, Hotel service providers and similar, Waiters, Gunsmiths, Sports, recreational and cultural service providers, Hairdressers, Beauticians, Traffic Police, State Police, Firemen, Private security guards
   8. ARTISTS: Artisans, Plumbers, Electricians, Tilers, Installers, Mechanics, Appliance Repairers, Goldsmiths, Decorators, Tailors, Bakers, Carpenters, Butchers, Fruit and Vegetable Makers
   9. SPECIALIZED WORKERS: Specialized workers
   10. FARMERS: Farmers and agricultural workers, Breeders, Fishermen
   11. MACHINE WORKERS AND DRIVERS: Industrial plant operators, founders, assembly line workers, general workers, drivers, carpenters
   12. UNSKILLED PROFESSIONS: Bailiffs, Doormen, Warehouse Keepers, Delivery Workers, Street Vendors, Litter Bins, Street Sweepers, Launderers, Garages, Farmers, Craftsmen and similar workers
   13. ARMED FORCES: Military of all orders and ranks
8. What is the type of dwelling occupied by your household?
   1. Own, fully paid
   2. Own, we are paying it
   3. For rent, sublet or leasing
   4. In usufruct
   5. Other form of tenure (untitled possession, de facto occupant, collective property, etc.)
9. What is the useful living area of your home? (It is understood by useful living area, that included within the exteriors of the house, including the common spaces).

_______________ squared meters ($m^{2}$).

1. How many people usually live in your household?
   1. Adult men (age 18 and above) [ ] persons
   2. Adult women (age 18 and above) [ ] persons
   3. Boy children (age 3 – 17) [ ] persons
   4. Girl children (age 3 – 17) [ ] persons
   5. Babies (boy) (age under 3) [ ] persons
   6. Babies (girl) (age under 3) [ ] persons
2. Are there other persons not living in your household because they are currently working away from home?
   1. Yes [ ] persons
   2. No
3. How many children are of school age in your household?
   1. [ ] children 3-11 y.o.
   2. [ ] children 12-18 y.o.
4. What behavior(s) have you adopted in response to COVID-19 outbreak? In case it applies, please select more than one item.
   1. Hand washing
   2. Limiting exiting home.
   3. Cover coughs/sneezes
   4. Cleaning surfaces daily
   5. Staying home when sick
   6. Working from home
   7. Nothing
   8. Wearing face mask
   9. Changing / cancelling travel plans
   10. Making family / communication plans
   11. Stocking up home supplies and medicine
5. How much would the following factors prevent you from fully isolating yourself?
6. Need to earn an income
   1. Very Unlikely
   2. Somewhat Unlikely
   3. Somewhat Likely
   4. Very Likely
7. Need to care for others outside your home, such as elderly parents
   1. Very Unlikely
   2. Somewhat Unlikely
   3. Somewhat Likely
   4. Very Likely
8. Don't want to miss certain social events / gatherings
   1. Very Unlikely
   2. Somewhat Unlikely
   3. Somewhat Likely
   4. Very Likely
9. Urge to practice sports
   1. Very Unlikely
   2. Somewhat Unlikely
   3. Somewhat Likely
   4. Very Likely
10. Need to leave the house for some time (for family tensions, psychological stress, boredom)
    1. Very Unlikely
    2. Somewhat Unlikely
    3. Somewhat Likely
    4. Very Likely
11. If you lose your job, for how long do you believe you could pay your bills?
    1. 1 month or less
    2. 2 -3 months
    3. 4 -5 months
    4. 6 months or more
12. Over the past week, have any of the following events happened to you?
13. Forced to stay at home (in shelter)
    1. Yes
    2. No
14. Lost your employment (job or livelihood)
    1. Yes
    2. No
15. Decrease in earning or income
    1. Yes
    2. No
16. Had to homeschool child(ren)
    1. Yes
    2. No
17. Unable to access health care when needed
    1. Yes
    2. No
18. Unable to get access to sufficient food
    1. Yes
    2. No
19. Sought to get tested for COVID-19
    1. Yes
    2. No
20. Had to fill application for unemployment subsidy or other government sponsored support
    1. Yes
    2. No
21. Sought help or support from charities or other non-governmental organizations
    1. Yes
    2. No
22. How is your health in general?
    1. Very good
    2. Good
    3. Neither good nor bad
    4. Bad
    5. Very bad
23. Do you have any long-standing illness or health problem?
    1. Yes
    2. No
24. Are you undergoing a long-term medical treatment?
    1. Yes
    2. No
25. Do you have or have you ever had any of the following health problems?
    1. Diabetes
    2. An allergy
    3. Asthma
    4. Hypertension (high blood pressure)
    5. Long-standing troubles with your muscles, bones and joints (rheumatism, arthritis)
    6. Cancer
    7. Cataract
    8. Migraine or frequent headaches
    9. Chronic bronchitis, emphysema
    10. Osteoporosis
    11. Stroke, cerebral hemorrhage
    12. Peptic ulcer (gastric or duodenal ulcer)
    13. Chronic anxiety or depression
26. Is someone close to you, currently experiencing long-term illness or disability?
    1. Yes
    2. No
27. Are you taking care of such a person?
    1. Yes
    2. No
28. As result of COVID-19 outbreak?
29. Have you visited a doctor?
    1. Yes
    2. No
30. Have you called a doctor and/or your health care center?
    1. Yes
    2. No
31. Have you contacted any phone number to reach the health authorities?
    1. Yes
    2. No
32. How many times did you visit a doctor during the last 12 months, before the COVID-19 outbreak?
    1. A few times
    2. Once
    3. Never
33. How often have you been bothered by the following over the past seven days?
    1. Felt down, depressed, or hopeless about the future
       1. Most or all the time (5-7 days)
       2. Occasionally or a moderate amount of time (3-4 days)
       3. Some or a little of the time (1-2) days
       4. Rarely or none of the time (less than 1 day)
    2. Felt little interest or pleasure in doing things
       1. Most or all the time (5-7 days)
       2. Occasionally or a moderate amount of time (3-4 days)
       3. Some or a little of the time (1-2) days
       4. Rarely or none of the time (less than 1 day)
    3. Felt nervous, anxious or on the edge
       1. Most or all the time (5-7 days)
       2. Occasionally or a moderate amount of time (3-4 days)
       3. Some or a little of the time (1-2) days
       4. Rarely or none of the time (less than 1 day)
    4. Had trouble falling or staying asleep, or sleeping too much
       1. Most or all the time (5-7 days)
       2. Occasionally or a moderate amount of time (3-4 days)
       3. Some or a little of the time (1-2) days
       4. Rarely or none of the time (less than 1 day)
    5. Felt bad about yourself — or that you are a failure or have let yourself or your family down
       1. Most or all the time (5-7 days)
       2. Occasionally or a moderate amount of time (3-4 days)
       3. Some or a little of the time (1-2) days
       4. Rarely or none of the time (less than 1 day)
    6. Had troubles concentrating on things
       1. Most or all the time (5-7 days)
       2. Occasionally or a moderate amount of time (3-4 days)
       3. Some or a little of the time (1-2) days
       4. Rarely or none of the time (less than 1 day)
    7. Had a physical reaction when thinking about the outbreak
       1. Most or all the time (5-7 days)
       2. Occasionally or a moderate amount of time (3-4 days)
       3. Some or a little of the time (1-2) days
       4. Rarely or none of the time (less than 1 day)
    8. Feeling tired or having little energy
       1. Most or all the time (5-7 days)
       2. Occasionally or a moderate amount of time (3-4 days)
       3. Some or a little of the time (1-2) days
       4. Rarely or none of the time (less than 1 day)
34. How has your wage/earnings been affected after the COVID-19 outbreak?
    1. No change, full pay
    2. Reduced pay
    3. My contract was terminated
    4. My business has closed temporarily or definitely
    5. Not paid by the company, government is subsidizing pay
    6. Not paid by company, employee takes unpaid leave
    7. Not paid, spending my savings/helped by acquaintances.
35. Has your employer taken any actions at the plants/offices as a result of the COVID-19 outbreak?
    1. Yes, closed plants/offices
    2. No, but monitoring closely
    3. No, they do not intend to close any facilities
36. Which of the following arrangements has your employer taken, or is taking, in response to the COVID-19 outbreak? In case it applies, please select more than one item.
    1. Disseminated protective gear (i.e., hand sanitizer, masks, gloves) for employees to use at their discretion
    2. Arranged for temperatures to be checked at the workplace
    3. Arranged special flexible working hours (i.e., reduced operating hours)
    4. Requested self-quarantine for employees who have travelled within the last 14 days
    5. Required self-quarantine for employees who have travelled within the last 14 days
    6. Cancelled all international travel
    7. Cancelled all domestic travel
    8. Cancelled only non-essential travel to countries where there are confirmed cases of the coronavirus
    9. Implemented a shift schedule to rotate staff and minimize the number of people at the worksite
    10. Addressed employee's psychological stress
    11. Conducted internal survey, interview or focus groups to understand what employees are thinking and feeling
    12. Captured informal information to better understand employee's state of mind
    13. Enhanced cleaning and sanitizing efforts across facilities
    14. Allowing employees to use their paid time off in whatever manner is most convenient to them
    15. Providing employees with a “work from home” playbook of best practices we know to be effective
    16. Distributed a series of checklists and FAQ’s regarding our company’s approach to health and wealth
    17. Established a private hotline for employees to alert the company to their potential infection in order to encourage self-disclosure

## **Section S1.2 The Questionnaire in Spanish**

**LIST EXPERIMENT**

Control

Abajo encontrará una lista de afirmaciones sobre las cuales algunas personas están de acuerdo y otras no están de acuerdo. Por favor, díganos con cuántas está de acuerdo. No necesitamos saber con CUÁLES, solo con CUÁNTAS.

1. La globalización ha beneficiado la mayoría de la población en el mundo
2. La inmigración es una amenaza para nuestro estilo de vida
3. El personal de salud confronta el mayor riesgo en esta pandemia
4. En asuntos importantes de políticas públicas, el gobierno tiene siempre que acatar la opinión de los expertos

Tratamiento uno

Abajo encontrará una lista de afirmaciones sobre las cuales algunas personas están de acuerdo y otras no están de acuerdo. Por favor, díganos con cuántas está de acuerdo. No necesitamos saber con CUÁLES, solo con CUÁNTAS.

1. La globalización ha beneficiado la mayoría de la población en el mundo
2. La inmigración es una amenaza para nuestro estilo de vida
3. El personal de salud confronta el mayor riesgo en esta pandemia
4. En asuntos importantes de políticas públicas, el gobierno tiene siempre que acatar la opinión de los expertos
5. Durante la pandemia, el gobierno no tiene que enfocarse solo en prevenir el contagio, sino en evitar una fuerte crisis económica

Tratamiento dos

Abajo encontrará una lista de afirmaciones sobre las cuales algunas personas están de acuerdo y otras no están de acuerdo. Por favor, díganos con cuántas está de acuerdo. No necesitamos saber con CUÁLES, solo con CUÁNTAS.

1. La globalización ha beneficiado la mayoría de la población en el mundo
2. La inmigración es una amenaza para nuestro estilo de vida
3. El personal de salud confronta el mayor riesgo en esta pandemia
4. En asuntos importantes de políticas públicas, el gobierno tiene siempre que acatar la opinión de los expertos
5. Durante la pandemia, el gobierno no debe solo comunicar a los ciudadanos qué hacer para adherir a las medidas de seguridad, sino también explicar claramente cómo está planeando la salida de la crisis

Tratamiento tres

Abajo encontrará una lista de afirmaciones sobre las cuales algunas personas están de acuerdo y otras no están de acuerdo. Por favor, díganos con CUÁLES está de acuerdo.

1. La globalización ha beneficiado la mayoría de la población en el mundo
2. La inmigración es una amenaza para nuestro estilo de vida
3. El personal de salud confronta el mayor riesgo en esta pandemia
4. En asuntos importantes de políticas públicas, el gobierno tiene siempre que acatar la opinión de los expertos
5. Durante la pandemia, el gobierno no tiene que enfocarse solo en prevenir el contagio, sino en evitar una fuerte crisis económica
6. Durante la pandemia, el gobierno no debe solo comunicar a los ciudadanos qué hacer para adherir a las medidas de seguridad, sino también explicar claramente cómo está planeando la salida de la crisis

**ENCUESTA LÍNEA BASE**

1. ¿Cuál es su edad?

__ años

1. ¿Cuál es su sexo?
   1. Femenino
   2. Masculino
   3. Otro
2. ¿Cuál es su máximo nivel de estudios finalizados?
   1. Primaria o menos
   2. Secundaria
   3. Algunos años de universidad o formación profesional de grado superior
   4. Universidad o formación profesional de grado superior
   5. Posgrado (Master, Doctorado, otra)
3. ¿Cuál es su estado civil?
   1. Soltero/a
   2. Casado/a o en unión civil
   3. Divorciado/a o viudo/a
4. ¿En qué franja sitúa su nivel de ingresos brutos? anuales en su hogar? familiar anual?
   1. 9.999 Euro o menos
   2. 10.000 Euro – 29.999 Euro
   3. 30.000 Euro – 49.999 Euro
   4. 50.000 Euro – 149.999 Euro
   5. 150.000 Euro o más
5. ¿Cuál de las siguientes opciones describe mejor su área de residencia?
   1. Urbana
   2. Suburbana
   3. Rural
6. ¿Cuál de las siguientes situaciones describe mejor su situación laboral actual ¿
   1. Activo
   2. En búsqueda de empleo
   3. Jubilado
   4. Otros (no trabaja/no busca trabajo/no estudia, responsable del hogar, discapacitado no trabajador)
   5. Estudiante
7. ¿Cuál de las siguientes ocupaciones describe mejor tu actividad principal?
   1. DIRECTORES: Directores, Ejecutivos, Directores de una empresa
   2. EMPRESARIOS, TITULARES DE ACTIVIDADES: Empresarios, pequeñas empresas o titulares de actividades
   3. PROFESIONALES INTELECTUALES, CIENTÍFICOS: Físicos, Químicos, Estadísticos, Informáticos, Ingenieros, Arquitectos, Biólogos, Veterinarios, Farmacéuticos, Médicos, Dentistas, Especialistas en ciencias de la gestión, comerciales y bancarias, Abogados, Procuradores, Notarios, Magistrados, Profesores universitarios (ordinarios y asociados), Especialistas en ciencias económicas, sociológicas, psicológicas, artísticas, políticas, filosóficas y literarias, Periodistas
   4. PROFESORES: Profesores de enseñanza secundaria superior e inferior
   5. PROFESIONALES TÉCNICOS: Técnicos físicos y químicos, Agentes de bolsa y cambio, Agentes comerciales, Representantes, Pilotos de avión y técnicos de aviación civil, Fotógrafos, Enfermeros, Comadronas, Dietistas, Higienistas, Paramédicos, Agentes de seguros, Expertos, Diseñadores, Trabajadores sociales informáticos, Ingenieros de construcción civil, Operadores de Web, Programadores
   6. EMPLEADOS: Empleados administrativos, personal de secretaría, contables, empleados en contacto directo con el público, cajeros, empleados de mostrador
   7. COMERCIANTES O SERVICIOS: Mayoristas y gerentes de ventas al por mayor y al por menor, Oficinistas y similares, Proveedores de servicios de hoteles y similares, Camareros, Armeros, Proveedores de servicios deportivos, recreativos y culturales, Peluqueros, Esteticistas, Policía de Tránsito, Policía Estatal, Bomberos, Guardias de seguridad privada
   8. ARTISTAS: Artesanos, Fontaneros, Electricistas, Alicatadores, Instaladores, Mecánicos, Reparadores de aparatos, Orfebres, Decoradores, Sastres, Panaderos, Carpinteros, Carniceros, Fruteros, Fruteros
   9. TRABAJADORES ESPECIALIZADOS: Trabajadores especializados
   10. AGRICULTORES: Agricultores y trabajadores agrícolas, Criadores, Pescadores
   11. TRABAJADORES Y CONDUCTORES DE MAQUINARIA: Operadores de plantas industriales, fundadores, trabajadores de líneas de ensamblaje, trabajadores en general, conductores, carpinteros
   12. PROFESIONES NO CUALIFICADAS: Alguaciles, Porteros, Almacenistas, Repartidores, Vendedores ambulantes, Papeleras, Barredores ambulantes, Lavanderas, Garajes, Campesinos, Artesanos y trabajadores asimilados
   13. FUERZAS ARMADAS: Militares de todas las órdenes y rangos.
8. La vivienda ocupada por su hogar es:
   1. De propiedad, totalmente pagada
   2. De propiedad, la estamos pagando
   3. En alquiler, subarriendo, o leasing
   4. En usufructo
   5. Otra forma de tenencia (posesión sin título, ocupante de hecho, propiedad
   6. colectiva, etc.)
9. ¿Cuál es la superficie útil habitable de su vivienda? (Se entiende por superficie útil habitable, la comprendida dentro de los muros exteriores de la vivienda, excluidos los espacios comunes).

_______________ metros cuadros ($m^{2}$).

1. ¿Cuántas personas viven normalmente en su hogar?
   1. Hombres mayores de edad (18 o más años) [ ] personas
   2. Mujeres mayores de edad (18 o más años) [ ] personas
   3. Niños (edad 3 – 17) [ ] personas
   4. Niñas (edad 3 – 17) [ ] personas
   5. Bebés (varones) (menor de 3 años) [ ] personas
   6. Bebés (mujeres) (menor de 3 años) [ ] personas
2. ¿Hay otras personas que no viven en este hogar porque están trabajando lejos de la casa en este momento?
   1. Si [ ] personas
   2. No
3. ¿Cuántos niños en edad escolar hay en este hogar?
   1. [ ] niños o niñas 3-11 años.
   2. [ ] adolescentes 12-18 años.
4. ¿Qué comportamientos ha adoptado como respuesta al brote de COVID-19? MULTIRESPUESTA – Seleccionar todas las que corresponda.
   1. Lavarse las manos
   2. Limitar las salidas de la casa
   3. Cubrir los accesos de tos/ estornudos
   4. Limpiar cada día las superficies
   5. Encerrarme en casa si enfermo
   6. Trabajar desde casa
   7. Ninguna
   8. El uso de mascarillas
   9. Cambiar / cancelar planes de viaje
   10. Hacer planes familiares
   11. Abastecerse de provisiones y medicamentos
5. ¿En qué medida los siguientes factores le impedirían aislarse completamente?
6. Necesidad de recibir un ingreso
   1. Muy poco probable
   2. Algo improbable
   3. Algo probable
   4. Muy probable
7. Necesidad de cuidar otras personas fuera de la casa, como parientes ancianos
   1. Muy poco probable
   2. Algo improbable
   3. Algo probable
   4. Muy probable
8. No querer faltar a ciertos eventos sociales / reuniones
   1. Muy poco probable
   2. Algo improbable
   3. Algo probable
   4. Muy probable
9. Deseo de practicar deporte
   1. Muy poco probable
   2. Algo improbable
   3. Algo probable
   4. Muy probable
10. Necesidad de abandonar la casa por un tiempo (por tensiones familiares, estrés psicológico, aburrimiento)
    1. Muy poco probable
    2. Algo improbable
    3. Algo probable
    4. Muy probable
11. Si pierde su trabajo, ¿por cuánto tiempo cree que podrá pagar sus recibos?
    1. 1 mes o menos
    2. 2-3 meses
    3. 4 -5 meses
    4. 6 meses o más
12. Durante la semana pasada, ¿le ha ocurrido alguno de los siguientes eventos?
13. Obligado a quedarse en casa o fuera de su propio hogar en un lugar de acogida
    1. Sí
    2. No
14. Perdió su empleo (trabajo o medio de vida)
    1. Sí
    2. No
15. Disminución de los ingresos
    1. Sí
    2. No
16. Tuvo que educar a los niños en la casa
    1. Sí
    2. No
17. Incapaz de acceder a la atención de la salud cuando es necesario
    1. Sí
    2. No
18. Incapaz de tener acceso a suficientes alimentos
    1. Sí
    2. No
19. Buscó realizarse la prueba de COVID-19
    1. Sí
    2. No
20. Tuvo que llenar una solicitud de subsidio de desempleo u otro tipo de apoyo patrocinado por el gobierno
    1. Sí
    2. No
21. Solicitó ayuda o apoyo a organizaciones de beneficencia u otras organizaciones no gubernamentales
    1. Sí
    2. No
22. ¿Cómo es su salud en general?
    1. Muy Buena
    2. Buena
    3. Ni Buena ni mala
    4. Mala
    5. Muy mala
23. ¿Tiene alguna enfermedad o problema de salud de larga duración?
    1. Sí
    2. No
24. ¿Está sometido a un tratamiento médico de larga duración?
    1. Sí
    2. No
25. ¿Tiene o ha tenido alguna vez algunos de los siguientes problemas de salud?
    1. Diabetes
    2. Alergia
    3. Asma
    4. Hipertensión (alta presión arterial)
    5. Problemas crónicos con tus músculos, huesos y articulaciones (reumatismos, artritis)
    6. Cáncer
    7. Catarata
    8. Migraña o dolor de cabeza frecuente
    9. Bronquitis crónica, enfisema
    10. Osteoporosis
    11. Apoplejía, hemorragia cerebral
    12. Úlcera péptica (úlcera gástrica o duodenal)
    13. Ansiedad crónica o depresión
26. ¿Hay alguien cercano a usted que esté experimentando actualmente una enfermedad de larga duración o una discapacidad?
    1. Sí
    2. No
27. ¿Se está ocupando de esa persona?
    1. Sí
    2. No
28. Como consecuencia del brote del COVID-19
29. ¿Ha visitado a un médico?
    1. Sí
    2. No
30. ¿Ha llamado a un médico y/o a su centro de salud?
    1. Sí
    2. No
31. ¿Ha contactado con algún número telefónico de las autoridades sanitarias?
    1. Sí
    2. No
32. ¿Cuántas veces ha acudido a citas médicas en los últimos 12 meses, antes del brote de COVID-19?
    1. Algunas veces
    2. Una
    3. Ninguna
33. ¿Con qué frecuencia se ha visto afectado por las siguientes situaciones en los últimos siete días?
    1. Se sentía bajo/a de ánimo, deprimido/a o sin esperanza
       1. La mayoría del tiempo (5-7 días)
       2. Ocasionalmente o un número limitado de veces (3-4 días)
       3. Algunas o muy pocas veces (1-2 días)
       4. Raramente o nunca (menos de un día)
    2. Se sentía con poco interés o encontraba poco placer en hacer las cosas
       1. La mayoría del tiempo (5-7 días)
       2. Ocasionalmente o un número limitado de veces (3-4 días)
       3. Algunas o muy pocas veces (1-2 días)
       4. Raramente o nunca (menos de un día)
    3. Se sentía nervioso/a, ansioso/a, o al límite
       1. La mayoría del tiempo (5-7 días)
       2. Ocasionalmente o un número limitado de veces (3-4 días)
       3. Algunas o muy pocas veces (1-2 días)
       4. Raramente o nunca (menos de un día)
    4. Tuvo dificultad para quedarse dormido/a, dificultad para mantenerse dormido/a o durmió más de lo usual
       1. La mayoría del tiempo (5-7 días)
       2. Ocasionalmente o un número limitado de veces (3-4 días)
       3. Algunas o muy pocas veces (1-2 días)
       4. Raramente o nunca (menos de un día)
    5. Se sintió mal consigo mismo, se sintió fracasado, o que se ha decepcionado a sí mismo o a su familia
       1. La mayoría del tiempo (5-7 días)
       2. Ocasionalmente o un número limitado de veces (3-4 días)
       3. Algunas o muy pocas veces (1-2 días)
       4. Raramente o nunca (menos de un día)
    6. Tuvo problemas de concentración o bien de olvidarse de las cosas
       1. La mayoría del tiempo (5-7 días)
       2. Ocasionalmente o un número limitado de veces (3-4 días)
       3. Algunas o muy pocas veces (1-2 días)
       4. Raramente o nunca (menos de un día)
    7. Tuvo una reacción física cuando pensó en el brote
       1. La mayoría del tiempo (5-7 días)
       2. Ocasionalmente o un número limitado de veces (3-4 días)
       3. Algunas o muy pocas veces (1-2 días)
       4. Raramente o nunca (menos de un día)
    8. Sintió cansancio o falta de energía
       1. La mayoría del tiempo (5-7 días)
       2. Ocasionalmente o un número limitado de veces (3-4 días)
       3. Algunas o muy pocas veces (1-2 días)
       4. Raramente o nunca (menos de un día)
34. ¿Cómo se ha visto afectado su salario/ganancias después del brote de COVID-19?
    1. Sin cambios, salario completo
    2. Reducción salarial
    3. Contrato rescindido, despedido
    4. Mi negocio ha cerrado temporal o definitivamente
    5. No remunerado por la empresa, el gobierno está subvencionando el salario
    6. No remunerado por la empresa, el empleado toma un permiso no remunerado Sin entrada de ingresos, gastando mis ahorros/ayudado por conocidos
35. ¿Ha tomado su empleador algunas medidas en las plantas/oficinas como resultado del brote de COVID-19?
    1. Sí, ha cerrado establecimientos/oficinas
    2. No, pero sigue controlando muy estrictamente
    3. No, no quieren cerrar las instalaciones
36. ¿Cuáles de las siguientes medidas ha tomado o está tomando su empleador, , en respuesta al brote de COVID-19? RESPUESTA MÚLTIPLE. Seleccionar todas las que corresponda.
    1. Distribuir Equipo de protección (es decir, desinfectante de manos, máscaras, guantes) para que los empleados lo utilicen a discreción
    2. Toma de temperatura en el lugar del trabajo Organizar un horario de trabajo flexible especial (es decir, un horario de trabajo reducido)
    3. Solicitud de autocuarentena para los empleados que han viajado en los últimos 14 día
    4. Autocuarentena obligatoria para los empleados que hayan viajado en los últimos 14 días
    5. Cancelación de todos los viajes internacionales
    6. Cancelación de todos los viajes nacionales
    7. Cancelación de sólo los viajes no esenciales a países donde hay casos confirmados de coronavirus
    8. Aplicación de un programa de turnos para rotar el personal y reducir al mínimo el número de personas en el lugar de trabajo
    9. Abordar el estrés psicológico del empleado
    10. Realizar una encuesta interna, entrevista o grupos focales para entender lo que los empleados están pensando y sintiendo
    11. Capturar información informal para comprender mejor el estado mental del empleado
    12. Intensificación de los esfuerzos de limpieza y saneamiento en todas las instalaciones
    13. Permitir a los empleados utilizar su tiempo libre remunerado de la manera que les resulte más conveniente
    14. Proporcionar a los empleados un procedimiento a seguir "trabajo desde casa" con las mejores prácticas que sabemos que son eficaces
    15. Distribución de una serie de listas de chequeo y preguntas frecuentes sobre el enfoque del empleador en materia de salud y bienestar
    16. Establecimiento de una línea telefónica privada para que los empleados alerten al empleador sobre su posible infección a fin de fomentar la prevención de futuros casos

## **Section S1.3 The questionnaire in Italian**

**LIST EXPERIMENT**

Gruppo di Controllo

Qui sotto vedrai una lista di affermazioni che alcune persone approvano, mentre altre no. Indica con quante affermazioni ti trovi d’accordo. Non abbiamo bisogno di sapere con QUALI ti trovi d’accordo, ma con QUANTE.

1. La maggior parte della popolazione del mondo ha beneficiato dalla globalizzazione
2. L'immigrazione è una minaccia per il nostro stile di vita
3. Gli operatori sanitari si trovano ad affrontare il rischio più grande in questa pandemia
4. Su importanti questioni di politica pubblica, il governo dovrebbe sempre seguire il parere degli esperti

T1 – Trattamento 1

Qui sotto vedrai una lista di affermazioni che alcune persone approvano, mentre altre no. Indica con quante affermazioni ti trovi d’accordo. Non abbiamo bisogno di sapere con QUALI ti trovi d’accordo, ma con QUANTE.

1. La maggior parte della popolazione del mondo ha beneficiato dalla globalizzazione
2. L'immigrazione è una minaccia per il nostro stile di vita
3. Gli operatori sanitari si trovano ad affrontare il rischio più grande in questa pandemia
4. Su importanti questioni di politica pubblica, il governo dovrebbe sempre seguire il parere degli esperti
5. Durante la pandemia, il governo non dovrebbe concentrarsi solo su come contenere il contagio, ma anche come evitare una crisi economica.

T2 - Trattamento 2

Qui sotto vedrai una lista di affermazioni che alcune persone approvano, mentre altre no. Indica con quante affermazioni ti trovi d’accordo. Non abbiamo bisogno di sapere con QUALI ti trovi d’accordo, ma con QUANTE.

1. La maggior parte della popolazione del mondo ha beneficiato dalla globalizzazione
2. L'immigrazione è una minaccia per il nostro stile di vita
3. Gli operatori sanitari si trovano ad affrontare il rischio più grande in questa pandemia
4. Su importanti questioni di politica pubblica, il governo dovrebbe sempre seguire il parere degli esperti
5. Durante la pandemia, il governo non dovrebbe concentrarsi solo sul comunicare ai cittadini come rispettare le misure di sicurezza, ma anche spiegare in modo chiaro come sta pianificando l’uscita dalla crisi.

T2 - Trattamento 3

Qui sotto vedrai una lista di affermazioni che alcune persone approvano, mentre altre no. Indica con QUALI affermazioni ti trovi d’accordo.

1. La maggior parte della popolazione del mondo ha beneficiato dalla globalizzazione
2. L'immigrazione è una minaccia per il nostro stile di vita
3. Gli operatori sanitari si trovano ad affrontare un rischio incredibile in questa pandemia
4. Su importanti questioni di politica pubblica, il governo dovrebbe sempre seguire il parere degli esperti
5. Durante la pandemia, il governo non dovrebbe concentrarsi solo su come contenere il contagio, ma anche come evitare una crisi economica.
6. Durante la pandemia, il governo non dovrebbe concentrarsi solo sul comunicare ai cittadini come rispettare le misure di sicurezza, ma anche spiegare in modo chiaro come sta pianificando l’uscita dalla crisi.
7. Quanti anni hai?

__

1. Di che sesso sei?
   1. Femminile
   2. Maschile
   3. Altro
2. Qual è il più alto grado di istruzione che hai ottenuto?
   1. Scuola elementare o meno
   2. Scuola media o scuola superiore
   3. Qualche anno di università (non completata)
   4. Laurea universitaria triennale
   5. Laurea universitaria magistrale/Dottorato/Altro
3. Qual è il tuo stato civile?
   1. Celibe/nubile mai sposato/a
   2. Sposato/sposata o In una coppia di fatto
   3. Divorziato/ divorziata o vedovo / vedova
4. Qual è il reddito annuale del tuo nucleo familiare?
   1. Sotto 9.999€
   2. 10.000€ – 29.999€
   3. 30.000€ – 49.999€
   4. 50.000€ – 149.999€
   5. Oltre 150.000€
5. Quale delle seguenti caratterizzazioni descrive meglio l’area della tua residenza?
   1. Urbana
   2. Suburbana
   3. Rurale
6. Quale tra le seguenti situazioni descrive meglio la tua posizione nel mercato del lavoro?
   1. Occupato/a
   2. In cerca di lavoro
   3. Pensionato/a
   4. Altro inattivo (non lavora-non cerca lavoro-non studia; oppure: casalinga, invalido non lavoratore, pensionato non da lavoro ecc.)
   5. Studente/ssa
7. Quale tra le seguenti attività descrive meglio la tua occupazione prevalente?
8. DIRIGENTI: Direttori, Dirigenti, Amministratori d’azienda
9. IMPRENDITORI, TITOLARI D’ATTIVITÀ: Imprenditori, Piccoli imprenditori o Titolari d’attività
10. PROFESSIONI INTELLETTUALI, SCIENTIFICHE: Fisici, Chimici, Statistici, Informatici, Ingegneri, Architetti, Biologi, Veterinari, Farmacisti, Medici, Dentisti, Specialisti delle scienze gestionali, commerciali e bancarie, Avvocati, Procuratori legali, Notai, Magistrati, Docenti universitari (ordinari e associati), Specialisti in scienze economiche, sociologiche, psicologiche, artistiche, politiche, filosofiche e letterarie, Giornalisti
11. INSEGNANTI: Professori di scuola secondaria superiore e inferiore
12. PROFESSIONI TECNICHE: Tecnici fisici, chimici, Agenti di borsa e di cambio, Agenti di commercio, Rappresentanti, Piloti di aereo e tecnici dell’aviazione civile, Fotografi, Infermieri, Ostetriche, Dietisti, Igienisti, Paramedici, Agenti assicurativi, Periti, Disegnatori, Assistenti sociali informatici, Elettrotecnici delle costruzioni civili, Operatori web, Programmatori
13. IMPIEGATI: Impiegati amministrativi, Personale di segreteria, Contabili, Impiegati a contatto diretto con il pubblico, Cassieri, Addetti allo sportello
14. COMMERCIANTI O ADDETTI AI SERVIZI: Esercenti e gestori delle vendite all’ingrosso e al dettaglio, Commessi e assimilati, Gestori di servizi alberghieri ed assimilati, Camerieri, Baristi, Addetti allo sport, al tempo libero, ai servizi ricreativi e culturali, Parrucchieri, Estetisti, Vigili urbani, Agenti della polizia di stato, Vigili del fuoco, Guardie private di sicurezza
15. ARTIGIANI: Artigiani, Idraulici, Elettricisti, Piastrellisti edili, Installatori, Meccanici, Riparatori di apparecchi, Orafi, Decoratori, Sarti, Panettieri, Falegnami, Macellai, Fruttivendoli
16. OPERAI SPECIALIZZATI: Operai specializzati
17. AGRICOLTORI: Agricoltori e operai agricoli, Allevatori, Pescatori
18. OPERAI E CONDUTTORI DI MACCHINARI: Conduttori di impianti industriali, Fonditori, Operai addetti alla catena di montaggio, Operaio generico, Autisti, Carpentieri
19. PROFESSIONI NON QUALIFICATE: Uscieri, Facchini, Magazzinieri, Fattorini, Venditori ambulanti, Bidelli, Spazzini, Lavandai, Garzoni, Braccianti agricoli, Manovali ed assimilati
20. FORZE ARMATE: Militari di ogni ordine e grado
21. In the tipo di abitazione vive il tuo nucleo familiare?
    1. Di proprietà, finita di pagare
    2. Di proprietà, stiamo ancora pagando il mutuo
    3. In affitto, sub-affitto, leasing
    4. In usufrutto
    5. Altre forme (occupante de facto, proprietà collettiva etc.)
22. Quant’è la superficie abitabile della propria abitazione (inclusi gli spazi esterni, come balconi e terrazze, e gli spazi comuni)?

____________ metri quadrati ($m^{2}$).

1. Quante persone di solito vivono nel tuo nucleo familiare?
   1. Adulti maschi (sopra 18 anni) [ ] persone
   2. Adulti femmine (sopra 18 anni) [ ] persone
   3. Bambini maschi (età 3 – 17) [ ] persone
   4. Bambine femmine (età 3 – 17) [ ] persone
   5. Neonati (maschi) (sotto 3 anni) [ ] persone
   6. Neonati (femmine) (sotto 3 anni) [ ] persone
2. Ci sono altre persone che non vivono nel vostro nucleo familiare perché al momento lavorano lontani da casa?
   1. Sì [ ] persone
   2. No
3. Quanti bambini/ragazzi sono in età scolastica nel vostro nucleo familiare?
   1. [ ] bambini dai 3 agli 11 anni
   2. [ ] ragazzi dai 12 ai 18 anni
4. Quali comportamenti hai adottato in risposta alla diffusione del COVID-19? Se rilevanti, seleziona anche più di un elemento.
   1. Lavarsi le mani
   2. Limitare uscite da casa
   3. Starnutire / tossire coprendomi con fazzoletto o con avambraccio
   4. Pulizia giornaliera delle superfici
   5. Rimanere a casa se ammalato
   6. Lavorare da casa
   7. Nessuno
   8. Indossare mascherina
   9. Cambiare/cancellare piani di viaggio
   10. Approntare piani per la famiglia e di comunicazione
   11. Accumulare cibo e medicine
5. In che misura i seguenti fattori ti impedirebbero di isolarti completamente a casa?
6. Bisogno di guadagnare uno stipendio
   1. Molto improbabile
   2. Abbastanza improbabile
   3. Abbastanza probabile
   4. Molto probabile
7. Bisogno di occuparmi di altri al di fuori della mia abitazione (es. genitori anziani)
   1. Molto improbabile
   2. Abbastanza improbabile
   3. Abbastanza probabile
   4. Molto probabile
8. Non perdermi eventi sociali
   1. Molto improbabile
   2. Abbastanza improbabile
   3. Abbastanza probabile
   4. Molto probabile
9. Bisogno di praticare sport
   1. Molto improbabile
   2. Abbastanza improbabile
   3. Abbastanza probabile
   4. Molto probabile
10. Bisogno di uscire di casa per un po’ di tempo (per tensioni familiari, stress psicologico, noia o altro)
    1. Molto improbabile
    2. Abbastanza improbabile
    3. Abbastanza probabile
    4. Molto probabile
11. Se perdi il tuo lavoro, per quanto credi che potresti essere in grado pagare le bollette?
    1. 1 mese o meno
    2. 2 -3 mesi
    3. 4 -5 mesi
    4. 6 mesi o più
12. Nell’ultima settimana, ti è mai capitato uno dei seguenti casi?
13. Forzato a stare a casa
    1. Si
    2. No
14. Perso il tuo impiego / lavoro / sussistenza
    1. Si
    2. No
15. Diminuzione dello stipendio o del guadagno
    1. Si
    2. No
16. Ho dovuto fare lezioni a casa ai miei bambini
    1. Si
    2. No
17. Non ho potuto accedere al servizio sanitario quando ho avuto bisogno
    1. Si
    2. No
18. Non sono riuscito ad avere abbastanza generi alimentari
    1. Si
    2. No
19. Chiamato per essere sottoposto al tampone per il COVID-19
    1. Si
    2. No
20. Effettuato una richiesta di sussidio di disoccupazione, cassa integrazione o altri sostegni finanziari statali
    1. Si
    2. No
21. Chiesto sostegno ad associazioni di volontariato o altre associazioni non governative
    1. Si
    2. No
22. Com’è la tua salute in generale?
    1. Molto buona
    2. Buona
    3. Né buona né cattiva
    4. Cattiva
    5. Molto cattiva
23. Hai una malattia cronica o qualche altro problema di salute di lungo corso?
    1. Si
    2. No
24. Sei attualmente sottoposto ad un trattamento medico a lungo termine?
    1. Si
    2. No
25. Hai attualmente o hai avuto uno dei seguenti problemi di salute?
    1. Diabete
    2. Allergia
    3. Asma
    4. Ipertensione (pressione alta)
    5. Problemi cronici ai muscoli, ossa o legamenti (reumatismi, artrite)
    6. Cancro
    7. Cataratta
    8. Emicranie o frequenti mal di testa
    9. Bronchiti croniche (enfisemia)
    10. Osteoporosi
    11. Infarto, emorragia cerebrale
    12. Ulcera (gastrica o duodenale)
    13. Ansia cronica o depressione
26. Qualcuno a te caro sta soffrendo al momento di qualche malattia cronica o disabilità?
    1. Si
    2. No
27. Ti stai prendendo cura di questa persona?
    1. Si
    2. No
28. Per quanto riguarda il contagio di COVID-19,
29. Sei stato da un dottore?
    1. Si
    2. No
30. Hai chiamato un dottore e/o un centro medico?
    1. Si
    2. No
31. Hai contattato il numero verde istituito dal servizio sanitario?
    1. Si
    2. No
32. Quante volte sei stato dal dottore negli ultimi 12 mesi, prima dell’emergenza COVID-19?
    1. Qualche volta
    2. Una volta
    3. Mai
33. Quante volte ti sei sentito in uno dei seguenti stati d’animo negli ultimi 7 giorni?
    1. Mi sono sentito depresso, o senza speranza nel futuro
       1. La maggior parte del tempo o sempre (5-7 giorni)
       2. Occasionalmente o per un discreto periodo di tempo (3-4 giorni)
       3. Qualche volta o poche volte (1-2 giorni)
       4. Raramente o mai (meno di 1 giorno)
    2. Ho sentito poco interesse o piacere nel fare le cose
       1. La maggior parte del tempo o sempre (5-7 giorni)
       2. Occasionalmente o per un discreto periodo di tempo (3-4 giorni)
       3. Qualche volta o poche volte (1-2 giorni)
       4. Raramente o mai (meno di 1 giorno)
    3. Mi sono sentito nervoso, ansioso, o sul punto di avere un esaurimento
       1. La maggior parte del tempo o sempre (5-7 giorni)
       2. Occasionalmente o per un discreto periodo di tempo (3-4 giorni)
       3. Qualche volta o poche volte (1-2 giorni)
       4. Raramente o mai (meno di 1 giorno)
    4. Ho avuto problemi ad addormentarmi o a dormire, o ad alzarmi dal letto
       1. La maggior parte del tempo o sempre (5-7 giorni)
       2. Occasionalmente o per un discreto periodo di tempo (3-4 giorni)
       3. Qualche volta o poche volte (1-2 giorni)
       4. Raramente o mai (meno di 1 giorno)
    5. Mi sono sentito male con me stesso — o che sono un fallimento o che ho abbandonato me stesso o la mia famiglia
       1. La maggior parte del tempo o sempre (5-7 giorni)
       2. Occasionalmente o per un discreto periodo di tempo (3-4 giorni)
       3. Qualche volta o poche volte (1-2 giorni)
       4. Raramente o mai (meno di 1 giorno)
    6. Ho avuto problemi a concentrarmi sulle cose, come leggere i giornali o guardare la televisione
       1. La maggior parte del tempo o sempre (5-7 giorni)
       2. Occasionalmente o per un discreto periodo di tempo (3-4 giorni)
       3. Qualche volta o poche volte (1-2 giorni)
       4. Raramente o mai (meno di 1 giorno)
    7. Ho avuto una reazione fisica quando pensavo al contagio
       1. La maggior parte del tempo o sempre (5-7 giorni)
       2. Occasionalmente o per un discreto periodo di tempo (3-4 giorni)
       3. Qualche volta o poche volte (1-2 giorni)
       4. Raramente o mai (meno di 1 giorno)
    8. Mi sono sentito stanco o ho avuto poca energia
       1. La maggior parte del tempo o sempre (5-7 giorni)
       2. Occasionalmente o per un discreto periodo di tempo (3-4 giorni)
       3. Qualche volta o poche volte (1-2 giorni)
       4. Raramente o mai (meno di 1 giorno)
34. Come è cambiato il tuo stipendio / guadagno dopo l’inizio dell’epidemia?
    1. Nessun cambiamento, continuo a percepire interamente le mie entrate
    2. Le mie entrate si sono ridotte
    3. Sono stato licenziato/a /il mio contratto non è stato rinnovato
    4. La mia attività ha chiuso temporaneamente o definitivamente
    5. Ricevo un sussidio dallo stato, non dall’azienda
    6. Non vengo pagato dall’azienda, e ho dovuto prendere ferie non pagate
    7. Non vengo pagato, spendo i miei risparmi / sono aiutato da conoscenti
35. Il tuo datore di lavoro ha fatto qualcosa riguardo le proprie sedi (fabbriche/uffici) dall’inizio della pandemia?
    1. Si, chiuso fabbriche/uffici
    2. No, ma sta monitorando costantemente
    3. No, non intendono chiudere nessuna sede
36. Quali delle seguenti misure o azioni il tuo datore di lavoro ha attuato, o sta attuando, dall’inizio della pandemia?
    1. Distribuito indumenti protettivi (es. mascherine, guanti, igienizzanti per mani) per i dipendenti da usare a loro discrezione
    2. Organizzato un controllo di temperatura (misurazione febbre) sul luogo di lavoro
    3. Organizzato speciali orari di lavoro flessibili (es. orari ridotti di lavoro)
    4. Richiesto quarantena volontaria per impiegati che hanno viaggiato negli ultimi 14 giorni
    5. Obbligato alla quarantena gli impiegati che hanno viaggiato negli ultimi 14 giorni
    6. Cancellato tutti i viaggi internazionali
    7. Cancellato tutti i viaggi nazionali
    8. Cancellato solo i viaggi non essenziali verso paesi dove ci sono casi confermati di COVID-19
    9. Introdotto dei turni di lavoro speciali per minimizzare il numero di lavoro presenti sul posto di lavoro
    10. Dato supporto agli impiegati che soffrono di stress psicologico
    11. Condotto sondaggi interni, interviste, focus group per capire cosa pensano e provano gli impiegati
    12. Raccolto informazioni in modo informale per capire meglio lo stato mentale degli impiegati
    13. Rinforzato gli sforzi di pulizie e di disinfestazione in diversi siti di lavoro
    14. Lasciato decidere agli impiegati come spendere le proprie ferie
    15. Distribuito una guida di pratiche virtuose su come lavorare al meglio da casa a tutti gli impiegati
    16. Distribuito una serie di check-list e FAQ’s (domande e risposte frequenti) sull’approccio che il tuo datore di lavoro ha adottato riguardo alla salute e le finanze
    17. Creato una linea telefonica speciale per gli impiegati per avvisare il datore di lavoro di potenziali contagi per incoraggiare le autodichiarazioni dei sintomi.

## **Section S1.4 Sample proportions**

|  | **Population** | | | | | |  | **Sample** | | | | | | |
| --- | --- | --- | --- | --- | --- | --- | --- | --- | --- | --- | --- | --- | --- | --- |
|  | **Female** | | | **Male** | | |  | **Female** | | | | **Male** | | |
| **Age** | 18-25 | 26-55 | 56-75 | 18-25 | 36-55 | 56-75 |  | 18-25 | 26-55 | 56-75 | 18-25 | | 36-55 | 56-75 |
| **Spain** | | | | | | | | | | | | | | |
| Northeast | 3.3% | 5.4% | 4.1% | 3.3% | 5.5% | 3.7% |  | 3.7% | 6.4% | 1.8% | 3.7% | | 5.2% | 2.0% |
| Madrid + Center | 3.4% | 5.6% | 4.0% | 3.5% | 5.6% | 3.7% |  | 5.5% | 7.3% | 1.3% | 5.5% | | 7.3% | 2.1% |
| Levante (Central East) | 1.7% | 2.8% | 2.1% | 1.8% | 2.9% | 1.9% |  | 2.4% | 4.2% | 1.5% | 2.8% | | 4.3% | 1.8% |
| North | 1.0% | 2.0% | 1.7% | 1.1% | 1.9% | 1.5% |  | 2.0% | 4.5% | 0.9% | 2.1% | | 4.1% | 1.6% |
| South | 3.7% | 5.7% | 3.8% | 3.8% | 5.8% | 3.6% |  | 2.6% | 4.0% | 0.9% | 3.1% | | 4.3% | 1.2% |
| **Italy** | | | | | | | | | | | | | | |
| Central Italy | 2.4% | 4.2% | 3.5% | 2.5% | 4.1% | 3.1% |  | 4.2% | 5.7% | 1.8% | 4.4% | | 4.3% | 2.5% |
| Islands | 1.5% | 2.2% | 1.9% | 1.6% | 2.2% | 1.7% |  | 1.9% | 2.7% | 0.9% | 1.2% | | 2.3% | 1.2% |
| Northeastern Italy + Northwestern | 5.5% | 9.5% | 8.0% | 5.8% | 9.5% | 7.3% |  | 7.7% | 11.7% | 3.5% | 7.0% | | 9.4% | 6.1% |
| Southern Italy | 3.2% | 4.7% | 3.9% | 3.4% | 4.5% | 3.6% |  | 4.3% | 5.0% | 1.3% | 3.7% | | 4.8% | 2.4% |
| **UK** | | | | | | | | | | | | | | |
| East of England + Midlands | 4.0% | 4.7% | 4.0% | 4.1% | 4.6% | 3.8% |  | 5.5% | 7.2% | 2.5% | 2.8% | | 5.6% | 2.8% |
| London | 2.7% | 2.7% | 1.5% | 2.7% | 2.7% | 1.4% |  | 5.1% | 3.0% | 0.8% | 5.1% | | 4.5% | 0.9% |
| North | 3.8% | 4.2% | 3.7% | 3.9% | 4.1% | 3.5% |  | 4.2% | 6.8% | 2.8% | 3.7% | | 7.3% | 2.6% |
| South | 3.3% | 4.2% | 3.7% | 3.4% | 4.0% | 3.5% |  | 2.8% | 3.9% | 2.9% | 1.8% | | 3.4% | 3.0% |
| Scotland + Wales | 2.5% | 2.9% | 2.6% | 2.6% | 2.8% | 2.4% |  | 1.1% | 1.8% | 1.3% | 0.9% | | 2.1% | 1.9% |

## **Section S2. Supplementary Statistical Analysis**

**Table S 1** Balancing of the covariates

| VARIABLES | Control | Treatment 1 | Treatment 2 | Treatment 3 | Balance test (Chi2) |
| --- | --- | --- | --- | --- | --- |
| Age | 41.94 (13.59) | 42.21 (13.94) | 41.77 (13.54) | 44.95 (13.57) | 0.048 |
| Female | 0.50 (0.50) | 0.48 (0.49) | 0.50 (0.50) | 0.55 (0.49) | 0.003 |
| Education | 3.20 (1.15) | 3.17 (1.13) | 3.19 (1.15) | 3.09 (1.14) | 0.004 |
| Married | 0.59 (0.49) | 0.59 (0.49) | 0.59 (0.49) | 0.61 (0.48) | 0.538 |
| Unemployed | 0.11 (0.31) | 0.13 (0.34) | 0.13 (0.34) | 0.11 (0.32) | 0.025 |
| Household income | 2.65 (0.99) | 2.61 (0.99) | 2.64 (1.00) | 2.50 (0.92) | 0.000 |
| Urban residency | 0.56 (0.49) | 0.55 (0.49) | 0.55 (0.49) | 0.53 (0.49) | 0.451 |
| Living area (m2) | 928.06 (25311) | 350.90 (3045) | 614.51 (8826) | 2734.76 (76309) | 0.627 |
| People in home | 3.11 (2.27) | 3.20 (3.39) | 3.16 (2.35) | 3.02 (2.64) | 0.268 |
| Children in school | 0.70 (1.58) | 0.71 (1.58) | 0.68 (1.20) | 0.62 (1.22) | 0.635 |
| Home ownership | 0.39 (0.48) | 0.40 (0.49) | 0.41 (0.49) | 0.39 (0.48) | 0.550 |

Note: This table reports the mean, the standard deviation (in parenthesis) between treatments and the Chi2 test over all treatments and control condition. ‘Age’ is the age of the respondent; this variable is constrained between 18-75. ‘Female’ is a dummy variable with a value of 1 if the respondent is female and 0 otherwise. ‘Education’ is a categorical variable with the categories described in Q3. ‘Married’ is a dummy a variable with a value of 1 if the respondent is married and 0 otherwise. ‘Unemployed’ is a dummy variable with a value of 1 if the respondent is in search of job and 0 otherwise. ‘Household income’ is a categorical variable with the categories described in Q5. ‘Urban residency’ is a dummy variable with the value of 1 if the respondent residency area is urban and 0 otherwise. ‘Living area’ reports the respondent home useful living area in $m^{2}$. ‘People in home’ reports how many people usually live in the respondent household. ‘Children in school’ reports how many children are of school age in the respondent household. ‘Homeownership’ is a dummy variable with the value of 1 if the respondent type of dwelling is own and fully paid.

**Table S 2** Statement “During the pandemic, the government should not focus only in preventing contagion but also in avoiding a major economic crisis”

|  | (1) | (2) | (3) | (4) |
| --- | --- | --- | --- | --- |
| VARIABLES | Total | Spain | UK | Italy |
|  | Number of items | | | |
|  |  |  |  |  |
| T1 | 0.62*** | 0.59*** | 0.60*** | 0.67*** |
|  | (0.02) | (0.04) | (0.04) | (0.04) |
| Age | 0.00*** | 0.00* | 0.01*** | 0.00 |
|  | (0.00) | (0.00) | (0.00) | (0.00) |
| UK | 0.06* | - | - | - |
|  | (0.03) | - | - | - |
| Italy | 0.09*** | - | - | - |
|  | (0.03) | - | - | - |
| Female | 0.01 | 0.03 | -0.01 | 0.04 |
|  | (0.02) | (0.04) | (0.04) | (0.04) |
| Education | 0.02 | 0.01 | -0.04** | 0.06*** |
|  | (0.01) | (0.02) | (0.02) | (0.02) |
| Married | 0.05* | 0.05 | 0.09* | 0.00 |
|  | (0.03) | (0.05) | (0.05) | (0.05) |
| Unemployed | -0.02 | -0.11* | 0.00 | 0.05 |
|  | (0.04) | (0.07) | (0.09) | (0.06) |
| 2 Household Income bracket | 0.06 | -0.02 | 0.05 | 0.15** |
|  | (0.05) | (0.08) | (0.09) | (0.07) |
| 3 Household Income bracket | 0.08* | -0.01 | 0.05 | 0.19** |
|  | (0.05) | (0.09) | (0.09) | (0.08) |
| 4 Household Income bracket | 0.12** | 0.00 | 0.21** | 0.11 |
|  | (0.05) | (0.10) | (0.09) | (0.09) |
| 5 Household Income bracket | 0.42*** | 0.79*** | 0.34** | 0.47*** |
|  | (0.09) | (0.20) | (0.15) | (0.14) |
| Suburban residency | 0.07** | 0.02 | 0.07 | 0.07 |
|  | (0.03) | (0.06) | (0.05) | (0.05) |
| Rural residency | 0.02 | -0.11 | 0.03 | 0.08 |
|  | (0.04) | (0.07) | (0.06) | (0.06) |
| Living area | -0.00 | 0.00 | -0.00 | 0.00 |
|  | (0.00) | (0.00) | (0.00) | (0.00) |
| People in home | -0.01* | -0.00 | -0.03*** | 0.00 |
|  | (0.01) | (0.01) | (0.01) | (0.01) |
| Children in school | -0.02** | -0.05*** | 0.01 | -0.02 |
|  | (0.01) | (0.01) | (0.01) | (0.03) |
| Home ownership | 0.02 | -0.02 | 0.05 | 0.03 |
|  | (0.03) | (0.05) | (0.05) | (0.04) |
| Constant | 2.06*** | 2.24*** | 2.23*** | 1.91*** |
|  | (0.08) | (0.13) | (0.14) | (0.13) |
|  |  |  |  |  |
| Observations | 6,314 | 2,102 | 2,112 | 2,100 |
| R-squared | 0.11 | 0.10 | 0.11 | 0.12 |
| F test | 41.32 | 15.59 | 17.26 | 19 |

Note: The dependent variable ‘Number of items’ describes the number of statements that the respondent agreed with in the list experiment. ‘T1’ is a dummy variable that has the value of 1 whether the respondent was assigned to the treatment 1 and 0 whether she was assigned to the control condition. ‘‘Age’ is the age of the respondent; this variable is constrained between 18-75. ‘UK’ is a dummy variable with the value of 1 if the respondent is from UK and 0 otherwise. ‘Italy’ is a dummy variable with the value of 1 if the respondent is from Italy and 0 otherwise. ‘Female’ is a dummy variable with a value of 1 if the respondent is female and 0 otherwise. ‘Education’ is a categorical variable with the categories described in Q3. ‘Married’ is a dummy a variable with a value of 1 if the respondent is married and 0 otherwise. ‘Unemployed’ is a dummy variable with a value of 1 if the respondent is in search of job and 0 otherwise. ‘2 Household Income bracket’ is a dummy variable with the value of 1 if the household income is between 10.000 Euro and 29.999 Euro and 0 otherwise. ‘3 Household Income bracket’ is a dummy variable with the value of 1 if the household income is between 30.000 Euro and 49.999 Euro and 0 otherwise. ‘4 Household Income bracket’ is a dummy variable with the value of 1 if the household income is between 50.000 Euro and 149.999 Euro and 0 otherwise. ‘5 Household Income bracket’ is a dummy variable with the value of 1 if the household income is 150.000 Euro or above and 0 otherwise. ‘Suburban residency’ is a dummy variable with the value of 1 if the respondent residency area is suburban and 0 otherwise. ‘Rural residency’ is a dummy variable with the value of 1 if the respondent residency area is rural and 0 otherwise. ‘Living area’ reports the respondent home useful living area in $m^{2}$. ‘People in home’ reports how many people usually live in the respondent household. ‘Children in school’ reports how many children are of school age in the respondent household. ‘Homeownership’ is a dummy variable with the value of 1 if the respondent type of dwelling is own and fully paid. Robust standard errors in parentheses *** p<0.01, ** p<0.05, * p<0.1.

**Table S 3** Statement “During the pandemic, the government should not only communicate citizens what to do to adhere to the safety measures, but also explain clearly how it is planning the way out”

|  | (1) | (2) | (3) | (4) |
| --- | --- | --- | --- | --- |
| VARIABLES | Total | Spain | UK | Italy |
|  | Number of items | | | |
|  |  |  |  |  |
| T2 | 0.70*** | 0.72*** | 0.73*** | 0.65*** |
|  | (0.02) | (0.04) | (0.04) | (0.04) |
| Age | 0.00*** | 0.01*** | 0.01*** | 0.00 |
|  | (0.00) | (0.00) | (0.00) | (0.00) |
| UK | 0.10*** | - | - | - |
|  | (0.03) | - | - | - |
| Italy | 0.03 | - | - | - |
|  | (0.03) | - | - | - |
| Female | -0.02 | -0.03 | -0.00 | -0.01 |
|  | (0.02) | (0.04) | (0.04) | (0.04) |
| Education | 0.04*** | 0.06*** | 0.00 | 0.05*** |
|  | (0.01) | (0.02) | (0.02) | (0.02) |
| Married | 0.03 | 0.04 | 0.10** | -0.02 |
|  | (0.03) | (0.05) | (0.05) | (0.05) |
| Unemployed | -0.05 | -0.12* | 0.05 | -0.04 |
|  | (0.04) | (0.06) | (0.08) | (0.06) |
| 2 Household Income bracket | 0.17*** | 0.17** | 0.09 | 0.23*** |
|  | (0.05) | (0.08) | (0.09) | (0.07) |
| 3 Household Income bracket | 0.19*** | 0.12 | 0.12 | 0.29*** |
|  | (0.05) | (0.09) | (0.09) | (0.08) |
| 4 Household Income bracket | 0.17*** | 0.12 | 0.22** | 0.07 |
|  | (0.05) | (0.10) | (0.09) | (0.09) |
| 5 Household Income bracket | 0.36*** | 0.13 | 0.24 | 0.57*** |
|  | (0.09) | (0.27) | (0.15) | (0.15) |
| Suburban residency | 0.02 | -0.08 | 0.08 | 0.01 |
|  | (0.03) | (0.06) | (0.05) | (0.05) |
| Rural residency | 0.02 | -0.09 | 0.06 | 0.04 |
|  | (0.04) | (0.08) | (0.06) | (0.06) |
| Living area | -0.00** | -0.00** | -0.00* | 0.00 |
|  | (0.00) | (0.00) | (0.00) | (0.00) |
| People in home | 0.02** | 0.02** | 0.00 | 0.02 |
|  | (0.01) | (0.01) | (0.01) | (0.01) |
| Children in school | -0.02** | -0.01 | -0.01 | -0.07* |
|  | (0.01) | (0.01) | (0.02) | (0.04) |
| Home ownership | 0.04 | 0.02 | 0.02 | 0.05 |
|  | (0.03) | (0.05) | (0.05) | (0.04) |
| Constant | 1.83*** | 1.71*** | 1.95*** | 1.94*** |
|  | (0.08) | (0.13) | (0.14) | (0.12) |
|  |  |  |  |  |
| Observations | 6,323 | 2,094 | 2,122 | 2,107 |
| R-squared | 0.13 | 0.14 | 0.15 | 0.13 |
| F test | 53.96 | 21 | 24.44 | 18.31 |

Note: The dependent variable ‘Number of items’ describes the number of statements that the respondent agreed with in the list experiment. ‘T2’ is a dummy variable that has the value of 1 whether the respondent was assigned to the treatment 2 and 0 whether she was assigned to the control condition. ‘Age’ is the age of the respondent; this variable is constrained between 18-75. ‘UK’ is a dummy variable with the value of 1 if the respondent is from UK and 0 otherwise. ‘Italy’ is a dummy variable with the value of 1 if the respondent is from Italy and 0 otherwise. ‘Female’ is a dummy variable with a value of 1 if the respondent is female and 0 otherwise. ‘Education’ is a categorical variable with the categories described in Q3. ‘Married’ is a dummy a variable with a value of 1 if the respondent is married and 0 otherwise. ‘Unemployed’ is a dummy variable with a value of 1 if the respondent is in search of job and 0 otherwise. ‘2 Household Income bracket’ is a dummy variable with the value of 1 if the household income is between 10.000 Euro and 29.999 Euro and 0 otherwise. ‘3 Household Income bracket’ is a dummy variable with the value of 1 if the household income is between 30.000 Euro and 49.999 Euro and 0 otherwise. ‘4 Household Income bracket’ is a dummy variable with the value of 1 if the household income is between 50.000 Euro and 149.999 Euro and 0 otherwise. ‘5 Household Income bracket’ is a dummy variable with the value of 1 if the household income is 150.000 Euro or above and 0 otherwise. ‘Suburban residency’ is a dummy variable with the value of 1 if the respondent residency area is suburban and 0 otherwise. ‘Rural residency’ is a dummy variable with the value of 1 if the respondent residency area is rural and 0 otherwise. ‘Living area’ reports the respondent home useful living area in $m^{2}$. ‘People in home’ reports how many people usually live in the respondent household. ‘Children in school’ reports how many children are of school age in the respondent household. ‘Homeownership’ is a dummy variable with the value of 1 if the respondent type of dwelling is own and fully paid. Robust standard errors in parentheses *** p<0.01, ** p<0.05, * p<0.1.

**Table S 4** Direct response

|  | (1) | (2) |
| --- | --- | --- |
| VARIABLES | Item 5 | Item 6 |
|  |  |  |
| Age | -0.00 | 0.00 |
|  | (0.00) | (0.00) |
| UK | -0.07 | -0.07* |
|  | (0.04) | (0.04) |
| Italy | 0.07* | -0.05 |
|  | (0.04) | (0.04) |
| Female | -0.03 | 0.03 |
|  | (0.03) | (0.03) |
| Education | -0.01 | 0.02 |
|  | (0.01) | (0.01) |
| Married | -0.01 | -0.00 |
|  | (0.03) | (0.03) |
| Unemployed | 0.05 | -0.09* |
|  | (0.05) | (0.05) |
| 2 Household Income bracket | 0.08 | 0.03 |
|  | (0.05) | (0.05) |
| 3 Household Income bracket | 0.08 | 0.01 |
|  | (0.06) | (0.06) |
| 4 Household Income bracket | 0.13** | 0.08 |
|  | (0.07) | (0.06) |
| 5 Household Income bracket | -0.15 | -0.04 |
|  | (0.14) | (0.15) |
| Suburban residency | -0.01 | -0.03 |
|  | (0.04) | (0.04) |
| Rural residency | -0.00 | -0.02 |
|  | (0.05) | (0.04) |
| Living area | 0.00*** | 0.00*** |
|  | (0.00) | (0.00) |
| People in home | -0.00 | 0.00 |
|  | (0.00) | (0.01) |
| Children in school | -0.01 | -0.04*** |
|  | (0.01) | (0.01) |
| Home ownership | -0.01 | 0.00 |
|  | (0.03) | (0.03) |
| Constant | 0.58*** | 0.57*** |
|  | (0.09) | (0.09) |
|  |  |  |
| Observations | 1,080 | 1,080 |
| R-squared | 0.02 | 0.02 |
| F test | 4.212 | 4.098 |

Note: The dependent variable ‘Item 5’ (‘Item 6’) is a dummy variable with the value of 1 if the respondent agreed with statement 5 (6) of the treatment 3 in the list experiment. ‘Age’ is the age of the respondent; this variable is constrained between 18-75. ‘UK’ is a dummy variable with the value of 1 if the respondent is from UK and 0 otherwise. ‘Italy’ is a dummy variable with the value of 1 if the respondent is from Italy and 0 otherwise. ‘Female’ is a dummy variable with a value of 1 if the respondent is female and 0 otherwise. ‘Education’ is a categorical variable with the categories described in Q3. ‘Married’ is a dummy a variable with a value of 1 if the respondent is married and 0 otherwise. ‘Unemployed’ is a dummy variable with a value of 1 if the respondent is in search of job and 0 otherwise. ‘2 Household Income bracket’ is a dummy variable with the value of 1 if the household income is between 10.000 Euro and 29.999 Euro and 0 otherwise. ‘3 Household Income bracket’ is a dummy variable with the value of 1 if the household income is between 30.000 Euro and 49.999 Euro and 0 otherwise. ‘4 Household Income bracket’ is a dummy variable with the value of 1 if the household income is between 50.000 Euro and 149.999 Euro and 0 otherwise. ‘5 Household Income bracket’ is a dummy variable with the value of 1 if the household income is 150.000 Euro or above and 0 otherwise. ‘Suburban residency’ is a dummy variable with the value of 1 if the respondent residency area is suburban and 0 otherwise. ‘Rural residency’ is a dummy variable with the value of 1 if the respondent residency area is rural and 0 otherwise. ‘Living area’ reports the respondent home useful living area in $m^{2}$. ‘People in home’ reports how many people usually live in the respondent household. ‘Children in school’ reports how many children are of school age in the respondent household. ‘Homeownership’ is a dummy variable with the value of 1 if the respondent type of dwelling is own and fully paid. Robust standard errors in parentheses *** p<0.01, ** p<0.05, * p<0.1.

**Table S 5** Frequencies – list experiment

| # List item | Control | T1 | T2 |
| --- | --- | --- | --- |
| Spain | | | |
| 0 | 1.5% | 1.2% | 1.4% |
| 1 | 13.6% | 7.7% | 6.5% |
| 2 | 43.4% | 25.3% | 19.7% |
| 3 | 32.3% | 36.8% | 37.5% |
| 4 | 9.2% | 21.6% | 27.2% |
| 5 | - | 7.5% | 7.8% |
| UK | | | |
| 0 | 1.1% | 1.8% | 1.0% |
| 1 | 10.1% | 6.4% | 4.0% |
| 2 | 41.5% | 19.5% | 17.9% |
| 3 | 37.9% | 39.0% | 39.0% |
| 4 | 9.3% | 24.5% | 30.0% |
| 5 | - | 8.7% | 7.9% |
| Italy | | | |
| 0 | 1.2% | 0.3% | 0.9% |
| 1 | 13.0% | 6.1% | 6.5% |
| 2 | 39.9% | 20.6% | 18.5% |
| 3 | 35.3% | 38.8% | 42.1% |
| 4 | 10.6% | 25.8% | 24.4% |
| 5 | - | 8.2% | 7.6% |
| Total | | | |
| 0 | 1.3% | 1.1% | 1.1% |
| 1 | 12.2% | 6.8% | 5.7% |
| 2 | 41.6% | 21.8% | 18.7% |
| 3 | 35.2% | 38.1% | 39.6% |
| 4 | 9.7% | 24.0% | 27.2% |
| 5 | - | 8.2% | 7.8% |

**Table S 6** Test ceiling and floor effects - treatment 1

|  | Coefficient | P>z |
| --- | --- | --- |
| Pr (R=0, S=1) | 0.01 | 0.7191 |
|  | (0.00) | 1.0000 |
| Pr (R=0, S=0) | 0.01 | 1.0000 |
|  | (0.01) | 1.0000 |
| Pr (R=1, S=1) | 0.05 | 1.0000 |
|  | (0.01) | 1.0000 |
| Pr (R=1, S=0) | 0.07 | 1.0000 |
|  | (0.01) | 1.0000 |
| Pr (R=2, S=1) | 0.25 | 1.0000 |
|  | (0.01) | 1.0000 |
| Pr (R=2, S=0) | 0.16 | 1.0000 |
|  | (0.01) | 1.0000 |
| Pr (R=3, S=1) | 0.22 | 1.0000 |
|  | (0.01) | 1.0000 |
| Pr (R=3, S=0) | 0.13 | 1.0000 |
|  | (0.01) | 1.0000 |
| Pr (R=4, S=1) | 0.08 | 1.0000 |
|  | (0.01) | 1.0000 |
| Pr (R=4, S=0) | 0.02 | 1.0000 |
|  | (0.01) | 0.9839 |

Note: Coefficient variable is the estimated probability of all possible types of item-count responses for treatment 1.

**Table S 7** Test for design effects - treatment 1

|  | K | Lambda | P>Lambda | #P>Lambda |
| --- | --- | --- | --- | --- |
| Pr (R, S=0) | 0 | 0.0000 | 1.0000 | 1.0000 |
| Pr (R, S=1) | 0 | 0.0000 | 1.0000 | 1.0000 |

Note: K is the number of estimated probabilities needed to be tested. The test uses the generalized moment selection procedure. #P indicates the Bonferroni-adjusted p-values.

**Table S 8** Test ceiling and floor effects – treatment 2

|  | Coefficient | P>z |
| --- | --- | --- |
| Pr (R=0, S=1) | 0.01 | 0.7626 |
|  | (0.00) | 1.0000 |
| Pr (R=0, S=0) | 0.01 | 1.0000 |
|  | (0.01) | 1.0000 |
| Pr (R=1, S=1) | 0.06 | 1.0000 |
|  | (0.01) | 1.0000 |
| Pr (R=1, S=0) | 0.05 | 1.0000 |
|  | (0.01) | 1.0000 |
| Pr (R=2, S=1) | 0.29 | 1.0000 |
|  | (0.01) | 1.0000 |
| Pr (R=2, S=0) | 0.11 | 1.0000 |
|  | (0.01) | 1.0000 |
| Pr (R=3, S=1) | 0.25 | 1.0000 |
|  | (0.01) | 1.0000 |
| Pr (R=3, S=0) | 0.09 | 1.0000 |
|  | (0.01) | 1.0000 |
| Pr (R=4, S=1) | 0.07 | 1.0000 |
|  | (0.01) | 1.0000 |
| Pr (R=4, S=0) | 0.01 | 1.0000 |
|  | (0.01) | 0.9969 |

Note: Coefficient variable is the estimated probability of all possible types of item-count responses for treatment 1.

**Table S 9** Test for design effects - treatment 2

|  | K | Lambda | P>Lambda | #P>Lambda |
| --- | --- | --- | --- | --- |
| Pr (R, S=0) | 0 | 0.0000 | 1.0000 | 1.0000 |
| Pr (R, S=1) | 0 | 0.0000 | 1.0000 | 1.0000 |

Note: K is the number of estimated probabilities needed to be tested. The test uses the generalized moment selection procedure. #P indicates the Bonferroni-adjusted p-values.

**Fig S 1** Spearman rank-order correlation between stress and conditional prediction

**Fig S 2** Out-of-bag error (OOB error) convergence

**Fig S 3** Comparing the cumulative distribution of exposure to susceptibility, vulnerability, behavioural response, predicted stress conditional (50% of training sample) and predicted stress conditional on economic vulnerability and negative economic shock.


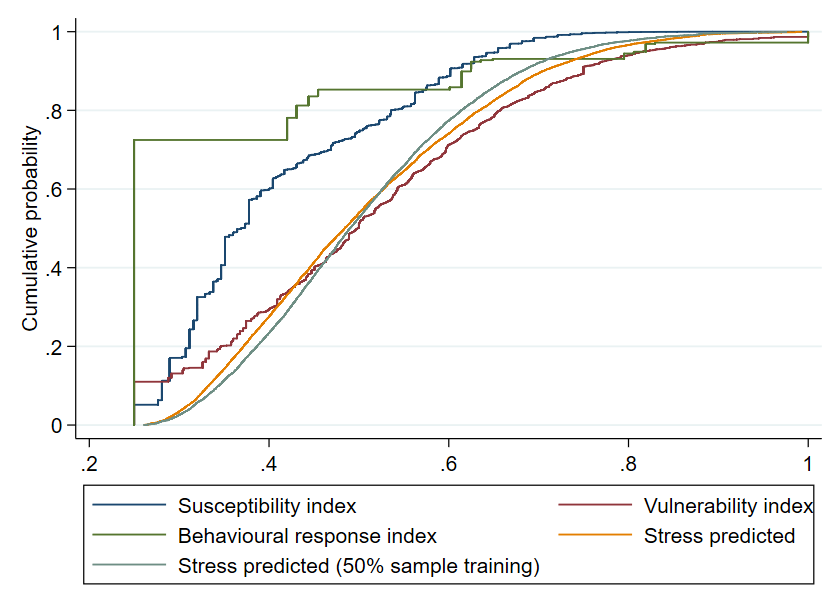


**Table S 10** Variable importance – random forest algorithm

| VARIABLES | IMPORTANCE |
| --- | --- |
| Household Income | 0.50 |
| Unemployed | 0.58 |
| Home ownership | 0.54 |
| Living area | 0.58 |
| People in home | 0.51 |
| Children in school | 0.53 |
| Cover the bills | 0.65 |
| Stress events | 0.96 |
| Income loss | 1.00 |

Note: This table reports the importance for each variable used in the random forest estimation. The values are scaled proportional to largest value in the set (i.e. ‘Income loss’). ‘Household income’ is a categorical variable with the categories described in Q5. ‘Unemployed’ is a dummy variable with a value of 1 if the respondent is in search of job and 0 otherwise. ‘Homeownership’ is a dummy variable with the value of 1 if the respondent’s type of dwelling is own and fully paid. ‘Living area’ reports the respondent home useful living area in $m^{2}$. ‘People in home’ reports how many people usually live in the respondent household. ‘Children in school’ reports how many children are of school age in the respondent household. ‘Cover the bills’ is a categorical variable with the categories described in Q16. ‘Stress events’ is an index composed by the questions Q17-a), b), c), d), e), f), h) & i). ‘Income loss’ is a dummy variable with the value of 1 if the respondent wage or earnings have been affected after the COVID-19 outbreak and 0 otherwise.

**Table S 11** Spearman correlation, total sample

|  | Stress | Stress predicted (15%) | Stress predicted (25%) | Stress predicted (33%) | Stress predicted (50%) |
| --- | --- | --- | --- | --- | --- |
| Stress predicted (15%) | 0.43*** | 1 |  |  |  |
| Stress predicted (25%) | 0.48*** | 0.57*** | 1 |  |  |
| Stress predicted (33%) | 0.54*** | 0.56*** | 0.57*** | 1 |  |
| Stress predicted (50%) | 0.65*** | 0.58*** | 0.60*** | 0.61*** | 1 |
| Stress predicted (100%) | 0.91*** | 0.55*** | 0.61*** | 0.66*** | 0.77*** |

**Table S 12** Spearman correlation, out-of-sample

|  | Stress | Stress predicted (15%) | Stress predicted (25%) | Stress predicted (33%) | Stress predicted (50%) |
| --- | --- | --- | --- | --- | --- |
| Stress predicted (15%) | 0.30*** | 1 |  |  |  |
| Stress predicted (25%) | 0.29*** | 0.62*** | 1 |  |  |
| Stress predicted (33%) | 0.30*** | 0.62*** | 0.65*** | 1 |  |
| Stress predicted (50%) | 0.30*** | 0.64*** | 0.66*** | 0.67*** | 1 |
| Stress predicted (100%) | 0.91*** | 0.45*** | 0.47*** | 0.49*** | 0.53*** |

**Table S13** Susceptibility to COVID-19

| VARIABLES | Spain | UK | Italy | Total |
| --- | --- | --- | --- | --- |
| Average age | 41 | 43 | 42 | 42 |
| At least one health problem | 62.0% | 56.8% | 57.4% | 58.7% |
| At least two health problems | 28.2% | 26.4% | 23.7% | 26.1% |
| At least one risky comorbidity | 29.0% | 33.2% | 27.6% | 29.9% |
| Bad or very bad health condition | 4.9% | 8.2% | 3.0% | 5.4% |
| Long-standing illness | 32.0% | 34.6% | 27.5% | 31.4% |
| Under long-term treatment | 29.2% | 24.9% | 21.6% | 25.2% |
| Visit a doctor during, more than once, the last 12 months | 59.0% | 42.9% | 58.0% | 53.3% |

Note: ‘Average age’ is the average age of the respondents in each country. ‘At least one health problem’ is the share of respondents that reported have or ever had at least one health problem in the list in Q21. ‘At least two health problems’ is the share of respondents that reported have or ever had at least two health problems in the list in Q21. ‘At least one risky comorbidity’ is the share of respondents that reported have or ever had diabetes, hypertension, asthma, a cardiovascular disease or cancer (Guan, et al. 2020; Yang et al. 2020; Richardson et al. 2020). ‘Bad or very bad health condition’ is the share of respondents that answered “bad or very bad” to Q18. ‘Long-standing illness’ is the share of respondents that answered “yes” to Q19. ‘Under long-term treatment’ is the share of respondents that answered “yes” to Q20. ‘Visit a doctor during, more than once, the last 12 months’ is the share of respondents that answered “a few times” to Q25.

**Table S 14** Vulnerability to COVID-19

| VARIABLES | Spain | UK | Italy | Total |
| --- | --- | --- | --- | --- |
| Need to earn an income | 53.6% | 48.7% | 66.4% | 56.2% |
| Need to care for others outside your home, such as elderly parents | 43.0% | 43.4% | 47.7% | 44.7% |
| Do not want to miss certain social events / gatherings | 21.3% | 15.2% | 19.0% | 18.5% |
| Urge to practice sports | 38.0% | 21.0% | 33.8% | 31.0% |
| Need to leave the house for some time (for family tensions, psychological stress, boredom) | 25.5% | 40.0% | 42.3% | 35.9% |

Note: This table report the share of respondents that answered “very likely” or “somewhat likely” to Q15- a), b), c), d) and e), respectively.

**Table S 15** Behavioural Response to COVID-19

| VARIABLES | Spain | UK | Italy | Total |
| --- | --- | --- | --- | --- |
| Sought to get tested for COVID-19 | 13.6% | 7.3% | 8.0% | 9.6% |
| As result of COVID-19 outbreak? Have you visited a doctor? | 12.8% | 5.9% | 11.0% | 9.9% |
| As result of COVID-19 outbreak? Have you called a doctor and/or your health care centre? | 26.3% | 13.9% | 16.0% | 18.7% |
| As result of COVID-19 outbreak? Have you contacted any phone number to reach the health authorities? | 19.2% | 12.2% | 9.6% | 13.7% |

Note: ‘Sought to get tested for COVID-19’ is the share of respondents that answered “Yes” to Q17- g). ‘As result of COVID-19 outbreak? Have you visited a doctor?’ is the share is the share of respondents that answered “Yes” to Q24- a). ‘As result of COVID-19 outbreak? Have you called a doctor and/or your health care centre?’ is the share of respondents that answered “Yes” to Q24- b). ‘As result of COVID-19 outbreak? Have you contacted any phone number to reach the health authorities?’ is the share of respondents that answered “Yes” to Q24- c).

**Table S 16** Principal component analysis - Kaiser–Meyer–Olkin (KMO)

| VARIABLES | KMO |
| --- | --- |
| Susceptibility index | 0.73 |
| Vulnerability index | 0.72 |
| Behavioural response index | 0.72 |

**Table S 17** Eigenvectors principal component analysis

| VARIABLES | Component 1 |
| --- | --- |
| Susceptibility index | |
| Bad or very bad health condition | 0.41 |
| Long-standing illness | 0.54 |
| Under long-term treatment | 0.51 |
| Comorbidity | 0.43 |
| Visit a doctor during, more than once, the last 12 months | 0.31 |
| Vulnerability index | |
| Need to earn an income | 0.41 |
| Need to care for others outside your home, such as elderly parents | 0.36 |
| Don't want to miss certain social events / gatherings | 0.49 |
| Urge to practice sports | 0.49 |
| Need to leave the house for some time (for family tensions, psychological stress, boredom) | 0.48 |
| Behavioural response | |
| Sought to get tested for COVID-19 | 0.43 |
| As result of COVID-19 outbreak? Have you visited a doctor? | 0.49 |
| As result of COVID-19 outbreak? Have you called a doctor and/or your health care centre? | 0.53 |
| As result of COVID-19 outbreak? Have you contacted any phone number to reach the health authorities? | 0.54 |

Note: ‘Bad or very bad health condition’ is the share of respondents that answered “bad or very bad” to Q18. ‘Long-standing illness’ is the share of respondents that answered “yes” to Q19. ‘Under long-term treatment’ is the share of respondents that answered “yes” to Q20. ‘Comorbidity’ is the sum of health problems reported by the respondent in the list in Q21. ‘Visit a doctor during, more than once, the last 12 months’ is the share of respondents that answered “a few times” to Q25. This vulnerability index is composed by the variables that report the respondents that answered “very likely” or “somewhat likely” to Q15- a), b), c), d) and e), respectively. ‘Sought to get tested for COVID-19’ is the share of respondents that answered “Yes” to Q17- g). ‘As result of COVID-19 outbreak? Have you visited a doctor?’ describes the percentage of respondents that answered “Yes” to Q24- a). ‘As result of COVID-19 outbreak? Have you called a doctor and/or your health care centre?’ is the share of respondents that answered “Yes” to Q24- b). ‘As result of COVID-19 outbreak? Have you contacted any phone number to reach the health authorities?’ is the share of respondents that answered “Yes” to Q24- c).
